# Supplementary material for: In vivo HIV-1 nuclear condensates safeguard against cGAS and license reverse transcription
Source: EMBO J. 2024 Dec 2;44(1):166–99. doi: 10.1038/s44318-024-00316-w (PMC11697293; doi:10.1038/s44318-024-00316-w)
Supplement: Supplementary file 1 — Appendix [file 44318_2024_316_MOESM1_ESM.pdf]

Appendix

In vivo HIV-1 nuclear condensates safeguard against cGAS and license reverse transcription

Selen Ay et al.

*Corresponding author: dinunzio@pasteur.fr

Contents

Appendix Table S1,S2,S3 1
Appendix Figure S1 2
Appendix Figure S2 3
Appendix Figure S3 4
Appendix Figure S4 5-7
Appendix Figure S5 8
Appendix Figure S6 9

Appendix Table S1.

qPCR primers for viral titration	
PRIMER NAME	PRIMER SEQUENCE
U5R forward	GGCTAACTAGGGAACCCACTG
U5R reverse	GCTAGAGATTTTCCACACTGACTAA
CD3 forward	GGCTATCATTCTTCTTCAAGGTA
CD3 reverse	CCTCTCTTCAGCCATTTAAGTA

Appendix Table S2.

PRIMARY AND SECONDARY smiFISH PROBES	
PROBE NAME	PROBE SEQUENCE
HIV1-01	GGGGATTGTAGGGAATTCCAAATTCCTGCTTTTACACTCGGACCTCGTCGACATGCATT
HIV1-02	CTTTTAGCTGACATTTATCACAGCTGGCTATTACACTCGGACCTCGTCGACATGCATT
HIV1-03	GTGTGCTGGTACCCATGCCAGATAGACTTACACTCGGACCTCGTCGACATGCATT
HIV1-04	AATACTGGAGTATTGTATGGATTTTCAGGCCCTTACACTCGGACCTCGTCGACATGCATT
HIV1-05	TTTTACTGGTACAGTCTCAATAGGGCTAATGGTTACACTCGGACCTCGTCGACATGCATT
HIV1-06	TATGTTGACAGGTGTAGGTCCTACTAATACTGTTACACTCGGACCTCGTCGACATGCATT
HIV1-07	CTAATCCTCATCCTGTCTACTTGCCATTACACTCGGACCTCGTCGACATGCATT
HIV1-08	CAATCATCACCTGCCATCTGTTTTCCATTTACACTCGGACCTCGTCGACATGCATT
HIV1-09	TTTCCAAAGTGGATTTCTGCTGTCCCTGTATTACACTCGGACCTCGTCGACATGCATT
HIV1-10	TTGTGGATGAATACTGCCATTTGTACTGCTGTTACACTCGGACCTCGTCGACATGCATT
HIV1-11	TTAAGATGTTCAGCCTGATCTCTTACCTGTTTACACTCGGACCTCGTCGACATGCATT
HIV1-12	TACAGTCTACTTGTCCATGCATGGCTTCTTACACTCGGACCTCGTCGACATGCATT
HIV1-13	TCATGTTCATCTTGGGCCTTATCTATTCCTTACACTCGGACCTCGTCGACATGCATT
HIV1-14	TGTCAGTTAGGGTGACAACTTTTTGTCTTCCTTTACACTCGGACCTCGTCGACATGCATT
HIV1-15	TGCTCCTACTATGGGTTCTTTCTCTAACTTTACACTCGGACCTCGTCGACATGCATT
HIV1-16	TCTGTTAGTGCTTTGGTTCCTCTAAGGAGTTTTTACACTCGGACCTCGTCGACATGCATT
HIV1-17	CTGTATGTCATTGACAGTCCAGCTGTCTTTTTTACACTCGGACCTCGTCGACATGCATT
HIV1-18	TGGCAGCACTATAGGCTGTACTGTCCTTACACTCGGACCTCGTCGACATGCATT
HIV1-19	TCTGATGTTTTTTGTCTGGTGTGGTAAGTCCCTTACACTCGGACCTCGTCGACATGCATT
HIV1-20	CCTCAACAGATGTTGTCTCAGCTCCTCTTACACTCGGACCTCGTCGACATGCATT
HIV1-21	ATTGCTGGTGATCCTTTCCATCCCTGTTACACTCGGACCTCGTCGACATGCATT
HIV1-22	TTTCTTTTTTAACCCTGCGGGATGTGGTATTCTTACACTCGGACCTCGTCGACATGCATT
HIV1-23	TTTAACTTTTGGGCCATCCATTCCTGGCTTACACTCGGACCTCGTCGACATGCATT
HIV1-24	CCCTATCTTTATTGTGACGAGGGGTCGTTGTTACACTCGGACCTCGTCGACATGCATT
Cy5FLAP	5'-[CY 5]A ATG CAT GTC GAC GAG GTC CGA GTC

Appendix Table S3.

qPCR primers	
PRIMER NAME	PRIMER SEQUENCE
ERT forward	GCCTCAATAAAGCTTGCCTTGA
ERT reverse	TGACTAAAAGGGTCTGAGGGATCT
LRT forward	TGTGTGCCCGTCTGTTGTGT
LRT reverse	GAGTCCTGCGTCGAGAGAGC
Actin forward	AACACCCCAGCCATGTACGT
Actin reverse	CGGTGAGGATCTTCATGAGGTAGT
LRT Probe	(FAM)-CAGTGGCGCCCGAACAGGGA-(TAMRA)

Appendix Figure S1

A

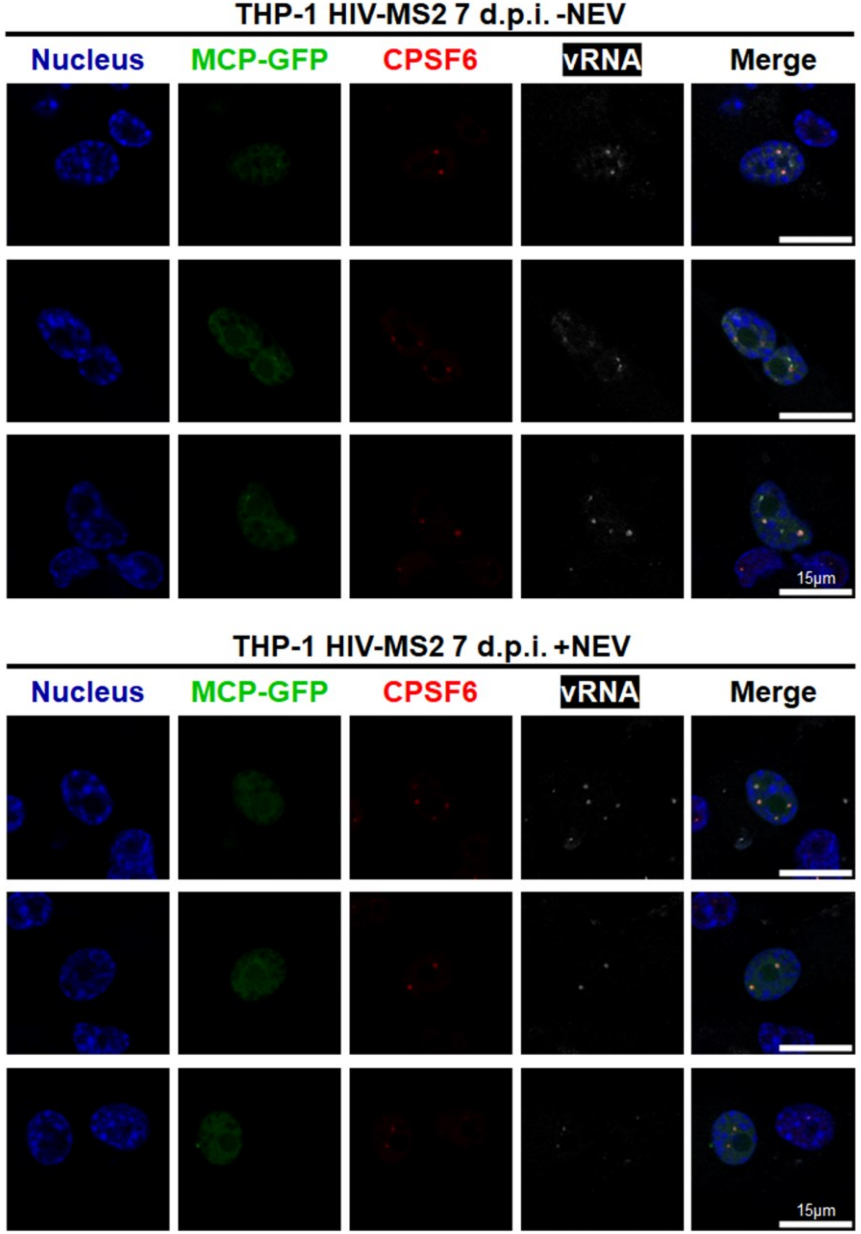

B

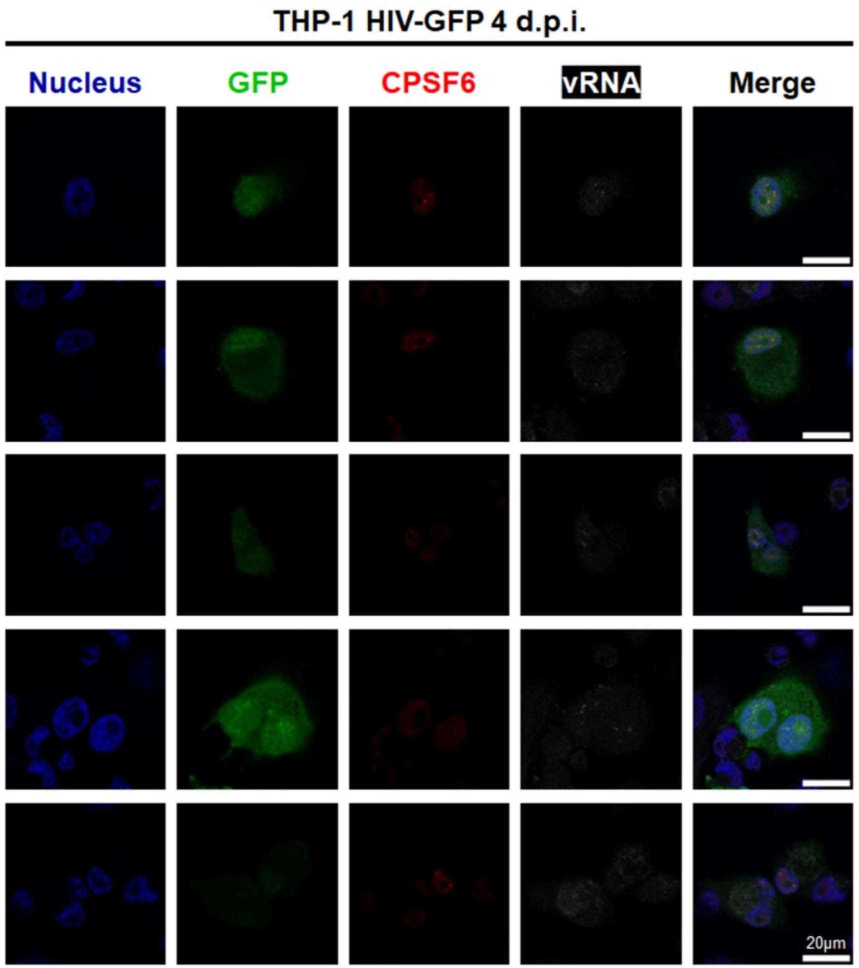

Multiple fields of view of THP-1 at 7 days post-infection +/-NEV and at 4days p.i. A) Multiple fields of view of Figure 2B (MCP-GFP (green), CPSF6 (red), RNA-FISH (gray), Hoechst (blue) scale bar=15µm. **B)** Multiple fields of view of Figure 2E (GFP (green), CPSF6 (red), RNA-FISH (gray), Hoechst (blue) scale bar=20µm.

Appendix Figure S2

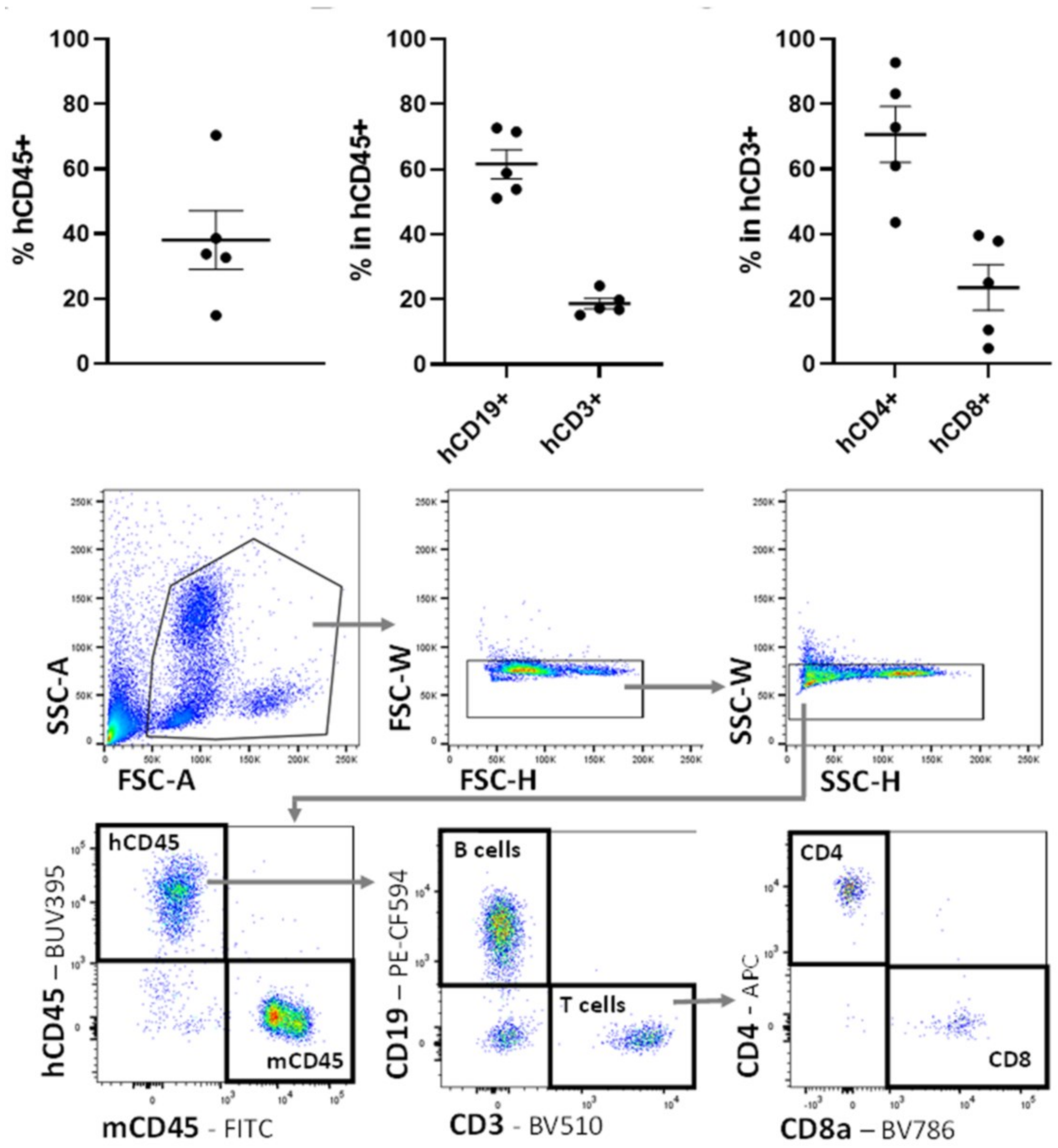

Human immune cell reconstitution in BRGSA2DR2 mice. Quantification of human immune cell populations in human immune system (HIS) mice. Top panels: in blood at 24 weeks post grafting, day -2 prior to HIV-1 NLAD8 infection. Percentage of hCD45 calculated as %hCD45 / (%hCD45 + %mCD45) (top, left panel), frequency of CD19+ B cells and CD3+ cells in hCD45 cells (top, middle panel), frequency of CD4+ and CD8+ in total CD3+ T cells (top, right panel) and gating strategy of blood cells (dot plots, FACS panels on the bottom).

Appendix Figure S3

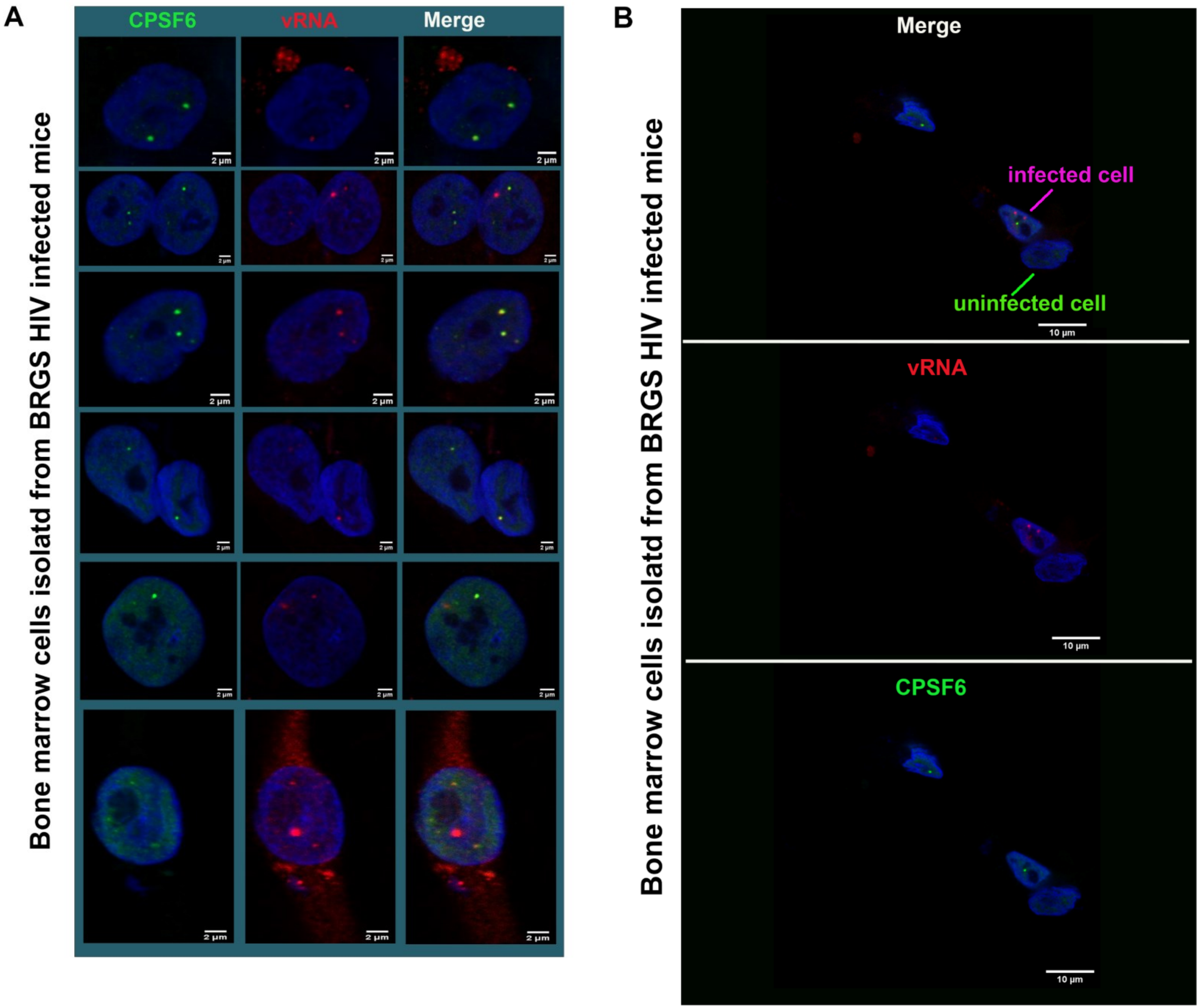

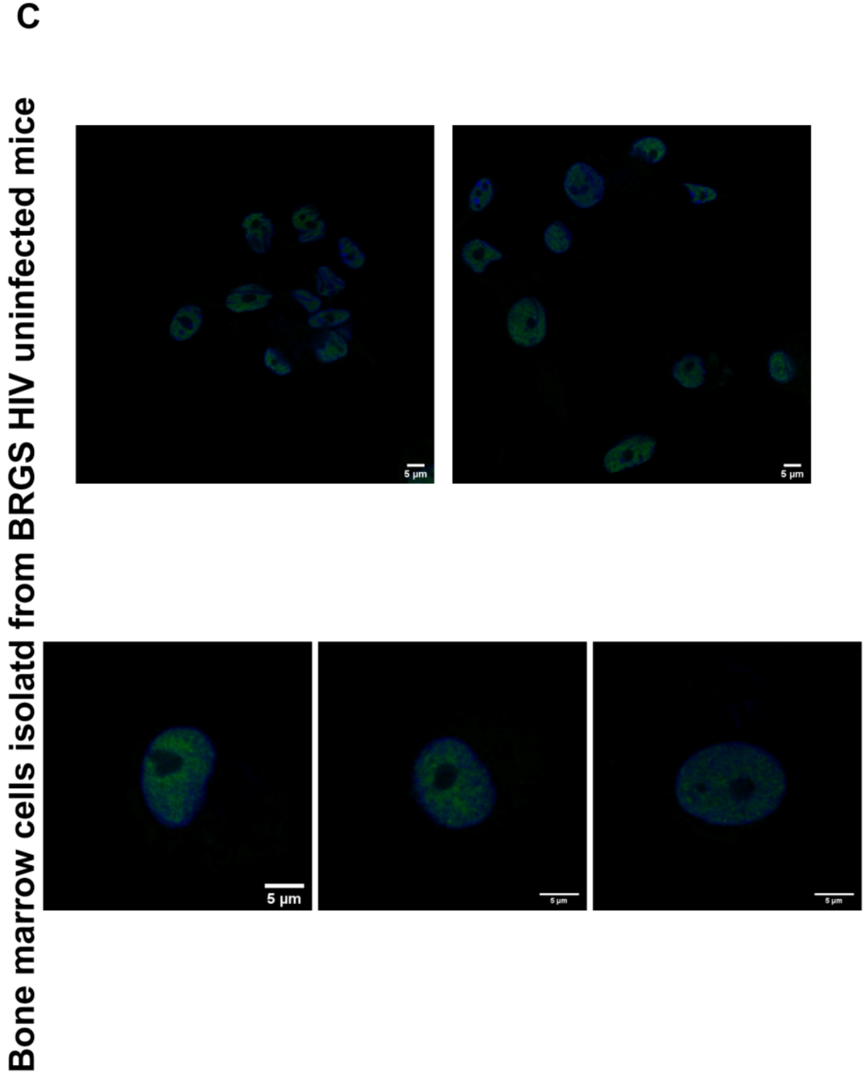

Staining of BM cells isolated from HIS mice infected with HIV-1. A-B) Immuno RNA-FISH in MDMs derived from the bone marrow of infected HIS mice. Viral RNA (vRNA) is in red and CPSF6 clusters in green. C) MDMs from uninfected mice.

Appendix Figure S4

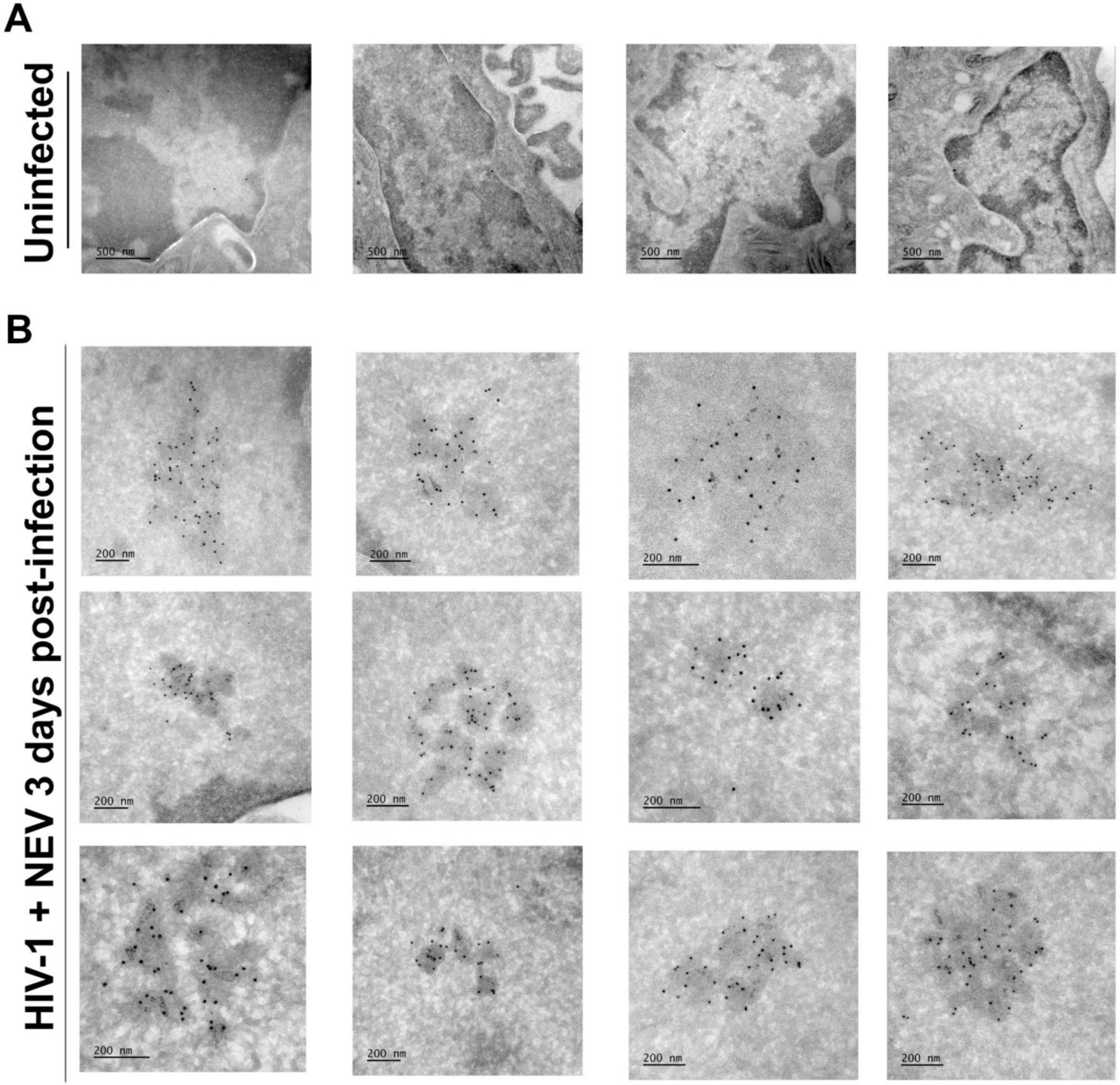

TEM projection images of Tokuyasu thin sections. A) uninfected THP-1 cells co-labelled with antibodies against CPSF6 and CA. B) Multiple sections labelled represent THP-1 cells at 3 days p.i. in presence of NEV.

Appendix Figure S4

C

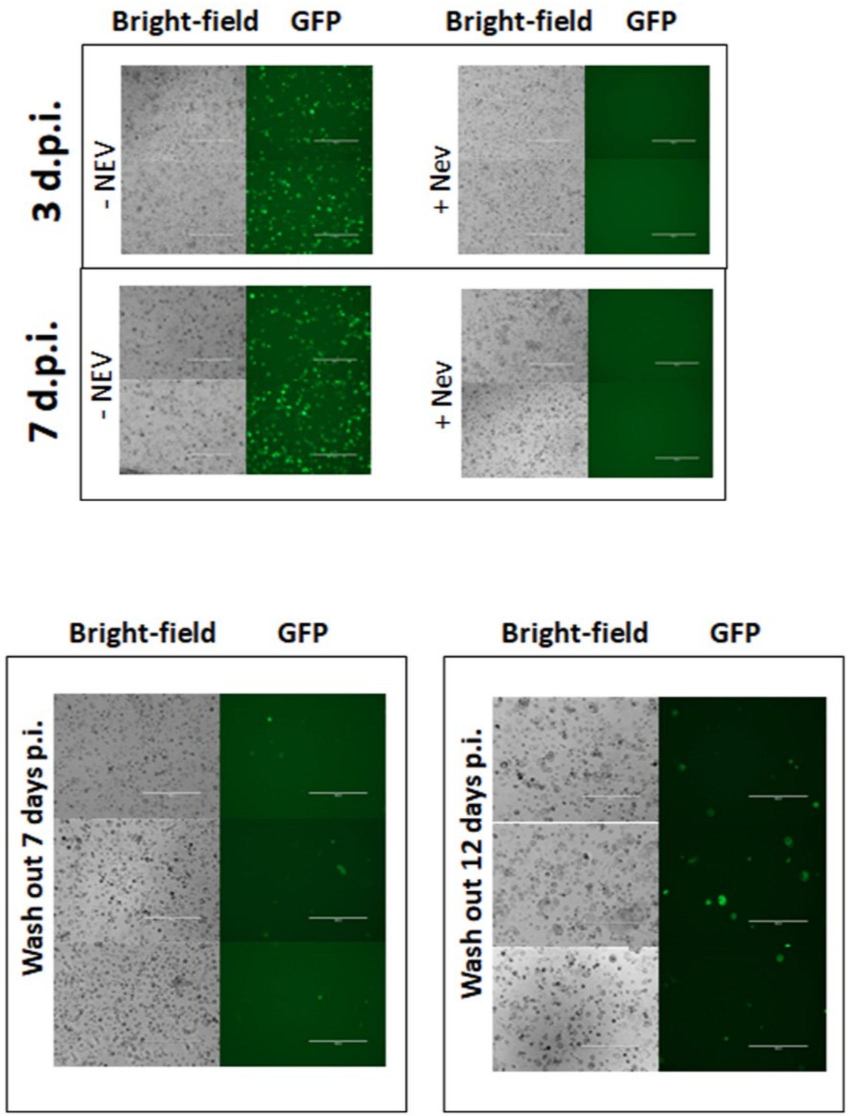

D

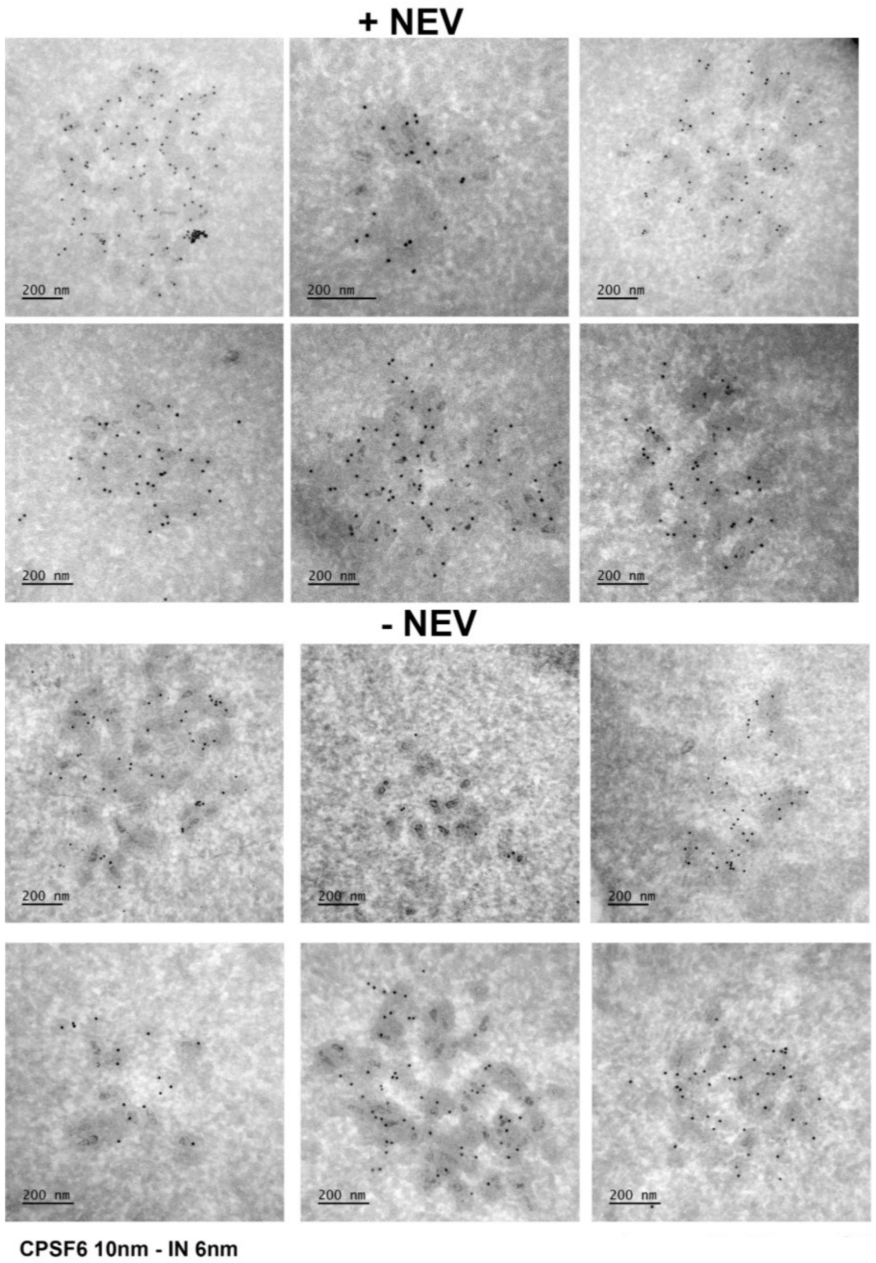

Asynchronous viral reactivation in nuclear HIV-1-MLOs.

C) THP-1 cells infected with HIV-1 ΔEnv pseudotyped with VSV-G and carrying the GFP as reporter gene at 3 and 7 d.p.i +/- NEV or after washout of NEV at 3 d.p.i. and live imaging (EVOS microscope) were done at 7 and 12 d.p.i., respectively. D) Sections of co-immuno-labeled (CPSF6 - 10nm gold and INHA-6nm gold) THP-1 cells infected for 7 days with NEV (top) and without NEV (bottom).

Appendix Figure S4

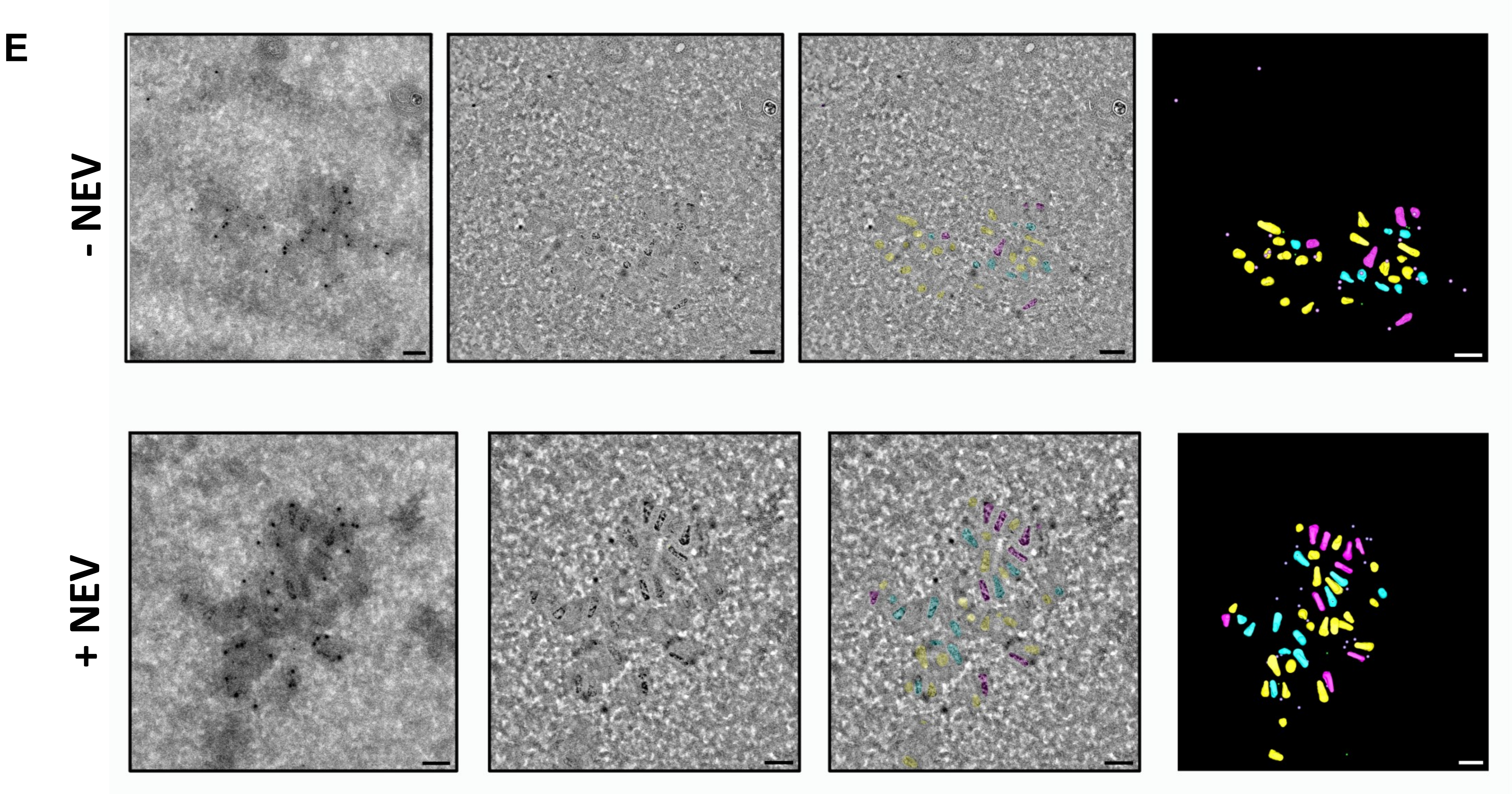

Structural characterization of the content of HIV-1-MLOs. Zero tilt projection images (left panels) on MLO areas, tomographic slices from the middle of the volume with and without pseudocoloring of the different core types (middle panels) and segmentation of cores (dense cores in magenta, lighter in cyan, ghosts in yellow) and immunogold beads (purple for CPSF6 and green for CA). Top panels -NEV and bottom panels +NEV.

Appendix Figure S5

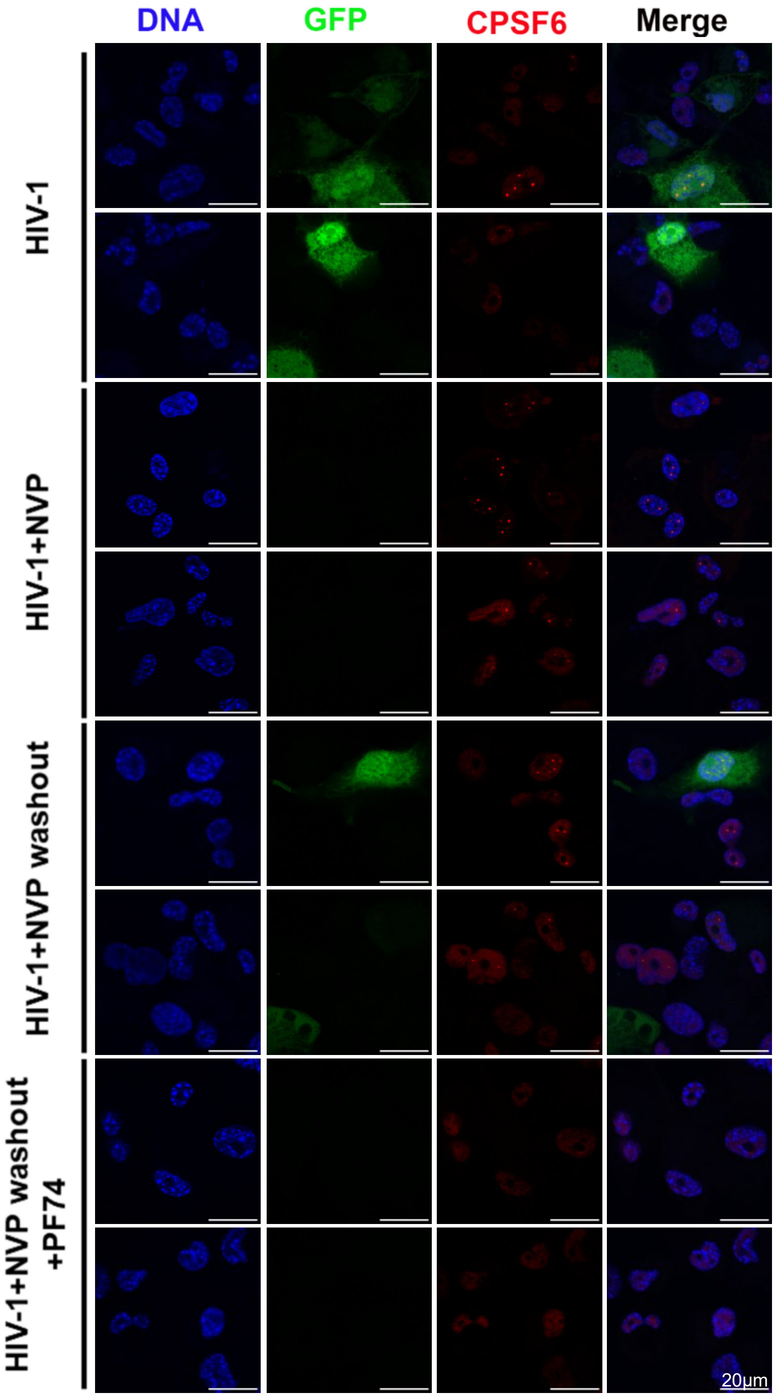

High PF74 dosage leads to the disassembly of CPSF6 condensates. Additional fields of view of cells kept in the same culture conditions as in Figure 5A (blue=Hoechst; green=GFP; red=CPSF6; scale bar=20µm).

Appendix Figure S6

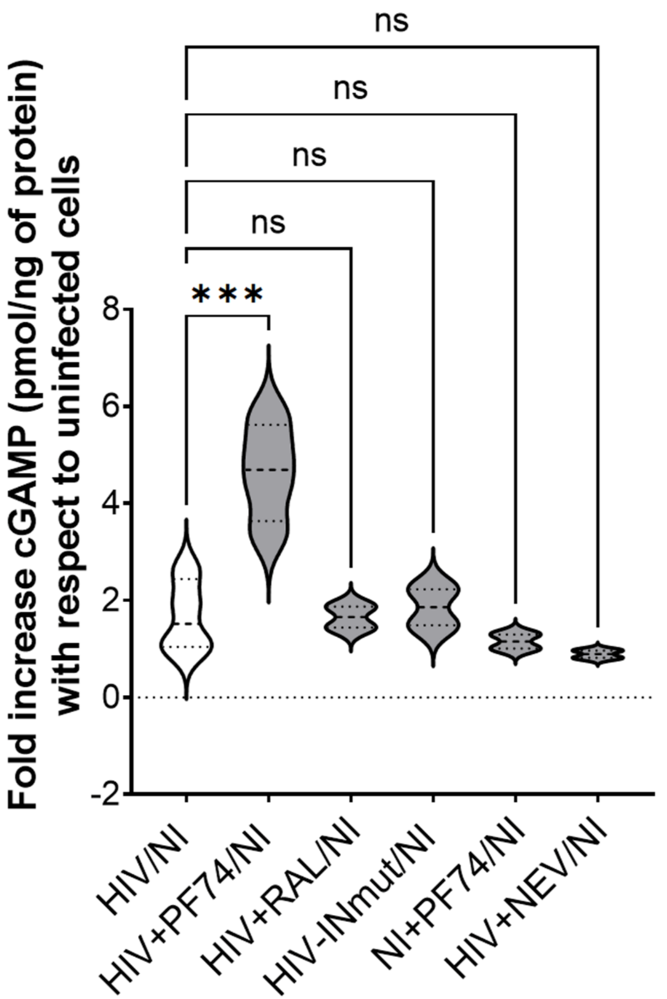

Fold increase in cGAMP. THP-1 cells were infected or not for 48h. Cell lysates were analyzed by ELISA to measure the production of cGAMP under several conditions: HIV infection, HIV infection + PF74 (10h.p.i.), HIV infection + raltegravir (RAL, an inhibitor of viral integration), infection with HIV carrying the IN mutant in the catalytic site, uninfected cells treated with PF74 for 10h, and HIV infection + nevirapine (NEV, an inhibitor of reverse transcription). The data are shown as the mean ± SD (Ordinary one-way ANOVA test; ns: p-value = 0.99, 0.99, 0.89, 0.66; *** denotes a p-value = 0.00076 .
