# Supplementary material for: In vivo HIV-1 nuclear condensates safeguard against cGAS and license reverse transcription
Source: EMBO J. 2024 Dec 2;44(1):166–99. doi: 10.1038/s44318-024-00316-w (PMC11697293; doi:10.1038/s44318-024-00316-w)
Supplement: Supplementary file 3 — Movie EV1 [file 44318_2024_316_MOESM3_ESM.zip › Movie EV1 legend.pdf]

Movie EV1. The THP1 cells were transduced with a lentiviral vector containing the coding sequence for the MCP-GFP fusion protein and then infected with HIV-1 MS2 for 7 days. The cells were fixed and labeled for the detection of CPSF6 (in red) and vRNA (in white). The MCP-GFP protein is seen in green, and the nuclei were stained with Hoechst (in blue). The 14 frames of the z-stack were acquired with a z-interval of 0.33µm.
