# Supplementary material for: In vivo HIV-1 nuclear condensates safeguard against cGAS and license reverse transcription
Source: EMBO J. 2024 Dec 2;44(1):166–99. doi: 10.1038/s44318-024-00316-w (PMC11697293; doi:10.1038/s44318-024-00316-w)
Supplement: Supplementary file 5 — Movie EV3 [file 44318_2024_316_MOESM5_ESM.zip › Movie EV3 legend.pdf]

Movie EV3. Movie mice silent cell: Mononucleated cells derived from BM of infected BRGS mice were differentiated ex-vivo and labeled for the detection of the vRNA (in red) and CPSF6 (in green). Nuclei were stained with Hoechst (in blue). The 12 frames of the z-stack were acquired with a z-interval of 0.33µm. (scale bar=2µm).
