# Supplementary material for: In vivo HIV-1 nuclear condensates safeguard against cGAS and license reverse transcription
Source: EMBO J. 2024 Dec 2;44(1):166–99. doi: 10.1038/s44318-024-00316-w (PMC11697293; doi:10.1038/s44318-024-00316-w)
Supplement: Supplementary file 6 — Movie EV4 [file 44318_2024_316_MOESM6_ESM.zip › Movie EV4 legend.pdf]

Movie EV4. Movie mice transcribing cell: Mononucleated cells derived from BM of infected BRGS mice were differentiated *ex-vivo* and labeled for the detection of the vRNA (in red) and CPSF6 (in green). Nuclei were stained with Hoechst (in blue). The 13 frames of the z-stack were acquired with a z-interval of 0.33 µm. (scale bar=2µm).
