# Supplementary material for: In vivo HIV-1 nuclear condensates safeguard against cGAS and license reverse transcription
Source: EMBO J. 2024 Dec 2;44(1):166–99. doi: 10.1038/s44318-024-00316-w (PMC11697293; doi:10.1038/s44318-024-00316-w)
Supplement: Supplementary file 7 — Movie EV5 [file 44318_2024_316_MOESM7_ESM.zip › Movie EV5 legend.pdf]

Movie EV5. The THP-1 cells were infected with HIV-1 for 7 days, sectioned and co-labeled with antibodies against CA and CPSF6. Dual-axis tilt series were collected in the same MLO area and a combined tomographic volume was finally produced. The CPSF6 was identified by gold particles of 10 nm, while the CA was identified by gold particles of 6 nm. Core-like shapes were annotated in different colors according to their type, dense cores in magenta, lighter cores in cyan, and ghosts in yellow. Gold beads that were used to detect CPSF6 were annotated using purple spheres of 10nm size, while gold beads that were used to detect the CA, were annotated with 6 nm spheres. A dense structure located near the ghost and could be the genetic material released from the viral core, which is now visible near a ghost (annotated in yellow).
