# Supplementary material for: In vivo HIV-1 nuclear condensates safeguard against cGAS and license reverse transcription
Source: EMBO J. 2024 Dec 2;44(1):166–99. doi: 10.1038/s44318-024-00316-w (PMC11697293; doi:10.1038/s44318-024-00316-w)
Supplement: Supplementary file 8 — Movie EV6 [file 44318_2024_316_MOESM8_ESM.zip › Movie EV6 legend.pdf]

Movie EV6. The THP-1 cells were infected with HIV-1 for 7 days with NEV, sectioned and co-labeled with antibodies against CA and CPSF6. Dual-axis tilt series were collected in the same MLO area and a combined tomographic volume was finally produced. The CPSF6 was identified by gold particles of 10 nm, while the CA was identified by gold particles of 6 nm. Core-like shapes were annotated with different colors according to their type, dense cores in magenta, lighter cores in cyan, and ghosts in yellow. Gold particles of 10 nm that were used to detect CPSF6, were annotated with 10nm in diameter purple spheres, while 6 nm gold beads that were used to detect the CA, were annotated with 6 nm green spheres.
