# Supplementary material for: In vivo HIV-1 nuclear condensates safeguard against cGAS and license reverse transcription
Source: EMBO J. 2024 Dec 2;44(1):166–99. doi: 10.1038/s44318-024-00316-w (PMC11697293; doi:10.1038/s44318-024-00316-w)
Supplement: Supplementary file 10 — Movie EV8 [file 44318_2024_316_MOESM10_ESM.zip › Movie EV8 legend.pdf]

Movie EV8. The tomographic data of movie 6 without annotations.
