# Supplementary material for: In vivo HIV-1 nuclear condensates safeguard against cGAS and license reverse transcription
Source: EMBO J. 2024 Dec 2;44(1):166–99. doi: 10.1038/s44318-024-00316-w (PMC11697293; doi:10.1038/s44318-024-00316-w)
Supplement: Supplementary file 11 — Movie EV9 [file 44318_2024_316_MOESM11_ESM.zip › Movie EV9 legend.pdf]

Movie EV9. Zoom view in the periphery of the Fig. 4F (upper panel) area without nevirapine, where a ghost core can be seen. The video scrolls through the XY planes of the tomographic volume. Dark material seems to have been released from the ghost core's head. CPSF6 immunogold beads are annotated in purple, CA immunogold beads in green. scale bar = 50 nm
