# Supplementary material for: In vivo HIV-1 nuclear condensates safeguard against cGAS and license reverse transcription
Source: EMBO J. 2024 Dec 2;44(1):166–99. doi: 10.1038/s44318-024-00316-w (PMC11697293; doi:10.1038/s44318-024-00316-w)
Supplement: Supplementary file 12 — Movie EV10 [file 44318_2024_316_MOESM12_ESM.zip › Movie EV10 legend.pdf]

Movie EV10. 3D surface representation of the ghost core seen in the periphery of the Fig. 4F (upper panel, without nevirapine). Isosurface representations for the ghost core internal (in yellow) and dark material released from the ghost core's head (in dark magenta). CPSF6 immunogold beads are annotated in purple and CA immunogold bead in green. Scale bar = 20 nm.
