# Supplementary material for: In vivo HIV-1 nuclear condensates safeguard against cGAS and license reverse transcription
Source: EMBO J. 2024 Dec 2;44(1):166–99. doi: 10.1038/s44318-024-00316-w (PMC11697293; doi:10.1038/s44318-024-00316-w)
Supplement: Supplementary file 13 — Movie EV11 [file 44318_2024_316_MOESM13_ESM.zip › Movie EV11 legend.pdf]

Movie EV11. Movie 5 with the 3D pixel classification predictions superimposed to IMOD contours that mark the HIV cones and to the IMOD points model that marks the positions of the immunogold. Magenta pixel predictions were based on the labelling of a dense core, blue pixel predictions on the labelling of a lighter core and yellow pixel predictions are based on the labelling of a ghost core. Manual tracing of HIV cones in IMOD follows the color coding of the pixel classification. CPSF6 immunogold is marked in purple, CA immunogold in green.
