# Supplementary material for: In vivo HIV-1 nuclear condensates safeguard against cGAS and license reverse transcription
Source: EMBO J. 2024 Dec 2;44(1):166–99. doi: 10.1038/s44318-024-00316-w (PMC11697293; doi:10.1038/s44318-024-00316-w)
Supplement: Supplementary file 14 — Movie EV12 [file 44318_2024_316_MOESM14_ESM.zip › Movie EV12 legend.pdf]

Movie EV12. Movie 6 with 3D pixel classification predictions superimposed to IMOD contours that mark the HIV cones. Magenta pixel predictions were based on the labelling of a dense core, blue pixel predictions on the labelling of a lighter core and yellow pixel predictions are based on the labelling of a ghost core. Manual tracing of HIV cones in IMOD follows the color coding of the pixel classification. Magenta and blue pixels outside cores are false positives generated due to the strong signal coming from the immunogold.
