# Supplementary figures and images for: In vivo HIV-1 nuclear condensates safeguard against cGAS and license reverse transcription
Source: EMBO J. 2024 Dec 2;44(1):166–99. doi: 10.1038/s44318-024-00316-w (PMC11697293; doi:10.1038/s44318-024-00316-w)

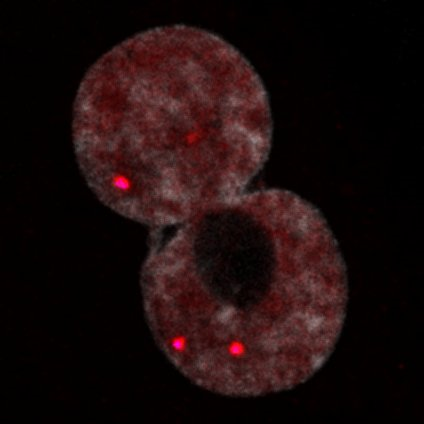

Supplement: Supplementary file 15 — Source data Fig. 1 [file 44318_2024_316_MOESM15_ESM.zip › Figure 1/1A/HIV1 - 11 days pi.tiff]

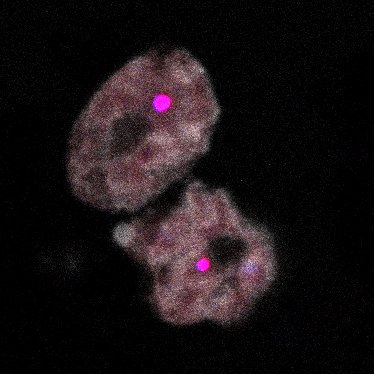

Supplement: Supplementary file 15 — Source data Fig. 1 [file 44318_2024_316_MOESM15_ESM.zip › Figure 1/1A/HIV1 - 18 days pi.tiff]

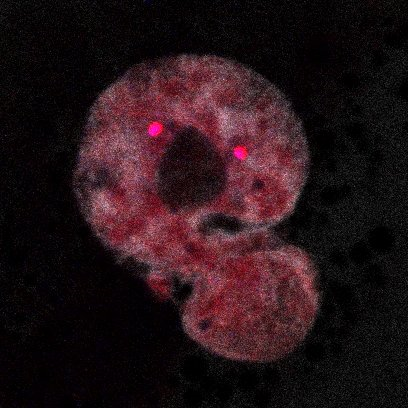

Supplement: Supplementary file 15 — Source data Fig. 1 [file 44318_2024_316_MOESM15_ESM.zip › Figure 1/1A/HIV1 - 25 days pi.tiff]

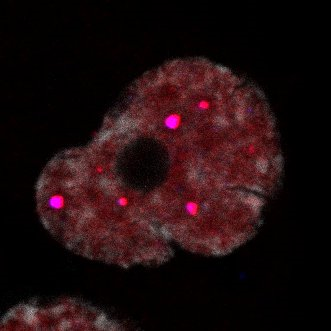

Supplement: Supplementary file 15 — Source data Fig. 1 [file 44318_2024_316_MOESM15_ESM.zip › Figure 1/1A/HIV1 - 4 days pi.tiff]

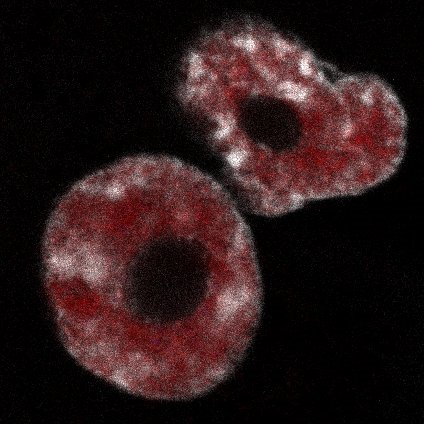

Supplement: Supplementary file 15 — Source data Fig. 1 [file 44318_2024_316_MOESM15_ESM.zip › Figure 1/1A/NI - 11 days pi.tiff]

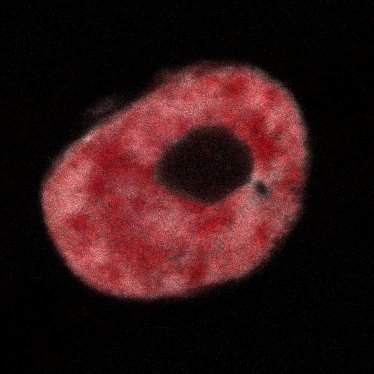

Supplement: Supplementary file 15 — Source data Fig. 1 [file 44318_2024_316_MOESM15_ESM.zip › Figure 1/1A/NI - 18 days pi.tiff]

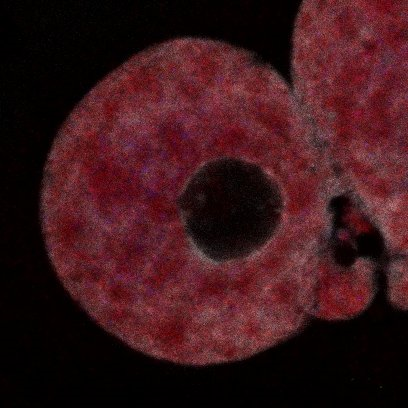

Supplement: Supplementary file 15 — Source data Fig. 1 [file 44318_2024_316_MOESM15_ESM.zip › Figure 1/1A/NI - 25 days pi.tiff]

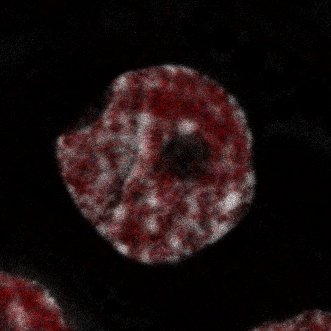

Supplement: Supplementary file 15 — Source data Fig. 1 [file 44318_2024_316_MOESM15_ESM.zip › Figure 1/1A/NI - 4 days pi.tiff]

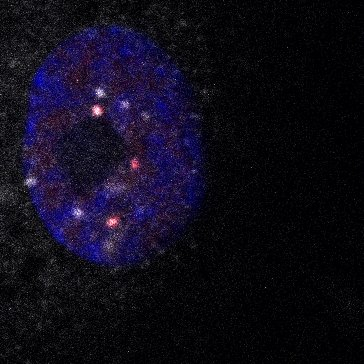

Supplement: Supplementary file 16 — Source data Fig. 2 [file 44318_2024_316_MOESM16_ESM.zip › Figure 2/2E/HIV1 4dpi vRNA CPSF6.tiff]

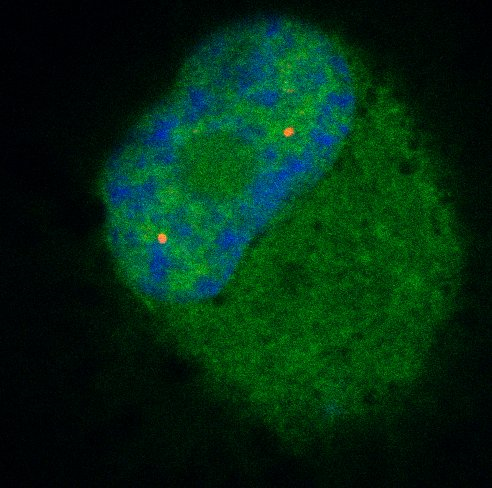

Supplement: Supplementary file 16 — Source data Fig. 2 [file 44318_2024_316_MOESM16_ESM.zip › Figure 2/2E/HIV1 4dpi GFP CPSF6.tiff]

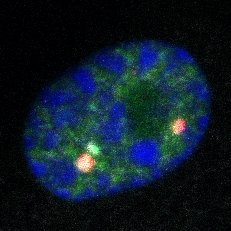

Supplement: Supplementary file 16 — Source data Fig. 2 [file 44318_2024_316_MOESM16_ESM.zip › Figure 2/2B/NEV- (neg).tiff]

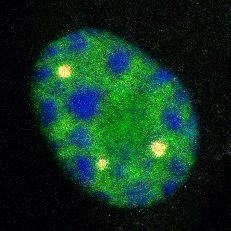

Supplement: Supplementary file 16 — Source data Fig. 2 [file 44318_2024_316_MOESM16_ESM.zip › Figure 2/2B/NEV+ (pos).tiff]

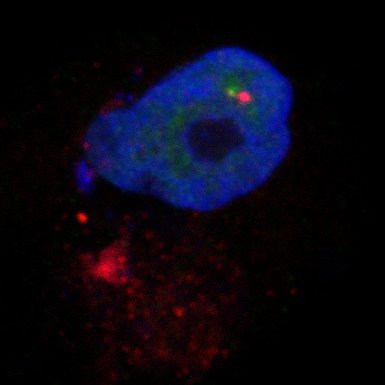

Supplement: Supplementary file 17 — Source data Fig. 3 [file 44318_2024_316_MOESM17_ESM.zip › Figure 3/3C/BM cell - Incoming vRNA and transcription foci.tiff]

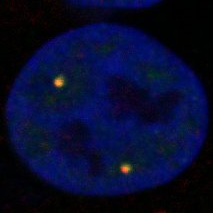

Supplement: Supplementary file 17 — Source data Fig. 3 [file 44318_2024_316_MOESM17_ESM.zip › Figure 3/3C/BM cell - Incoming vRNA.tiff]

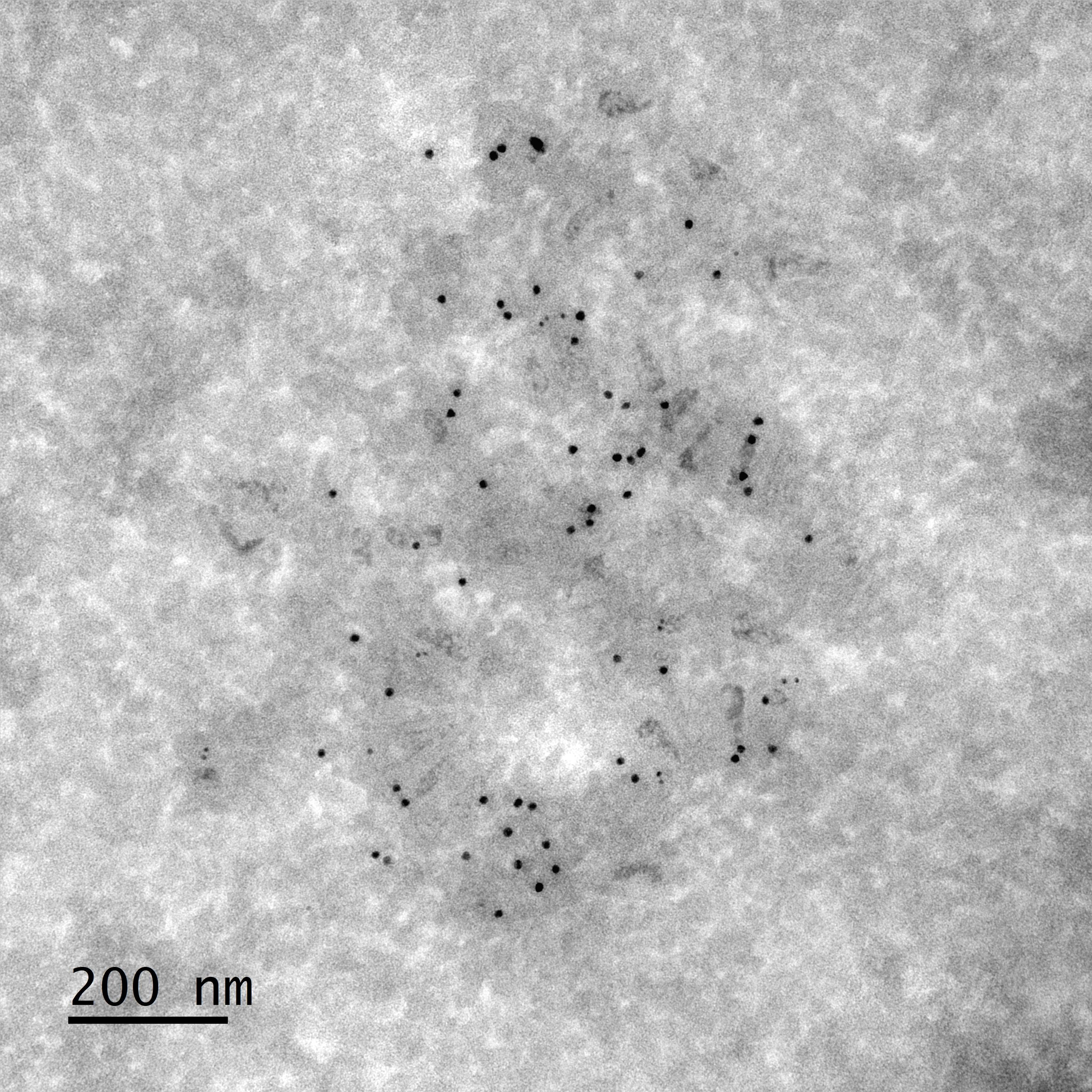

Supplement: Supplementary file 18 — Source data Fig. 4 [file 44318_2024_316_MOESM18_ESM.zip › Figure 4/Figure 4D/HIV -NEV.tif]

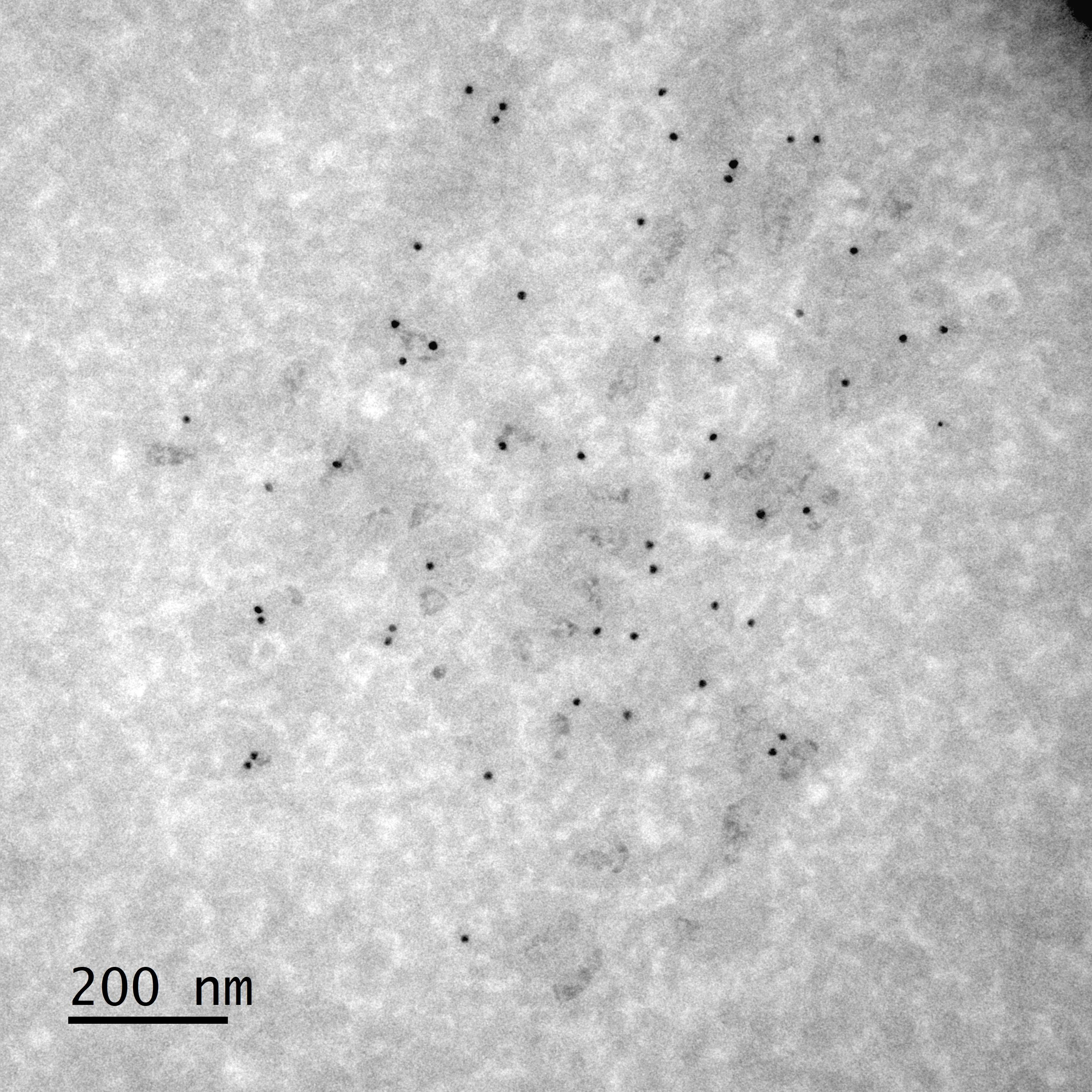

Supplement: Supplementary file 18 — Source data Fig. 4 [file 44318_2024_316_MOESM18_ESM.zip › Figure 4/Figure 4D/HIV +NEV.tif]

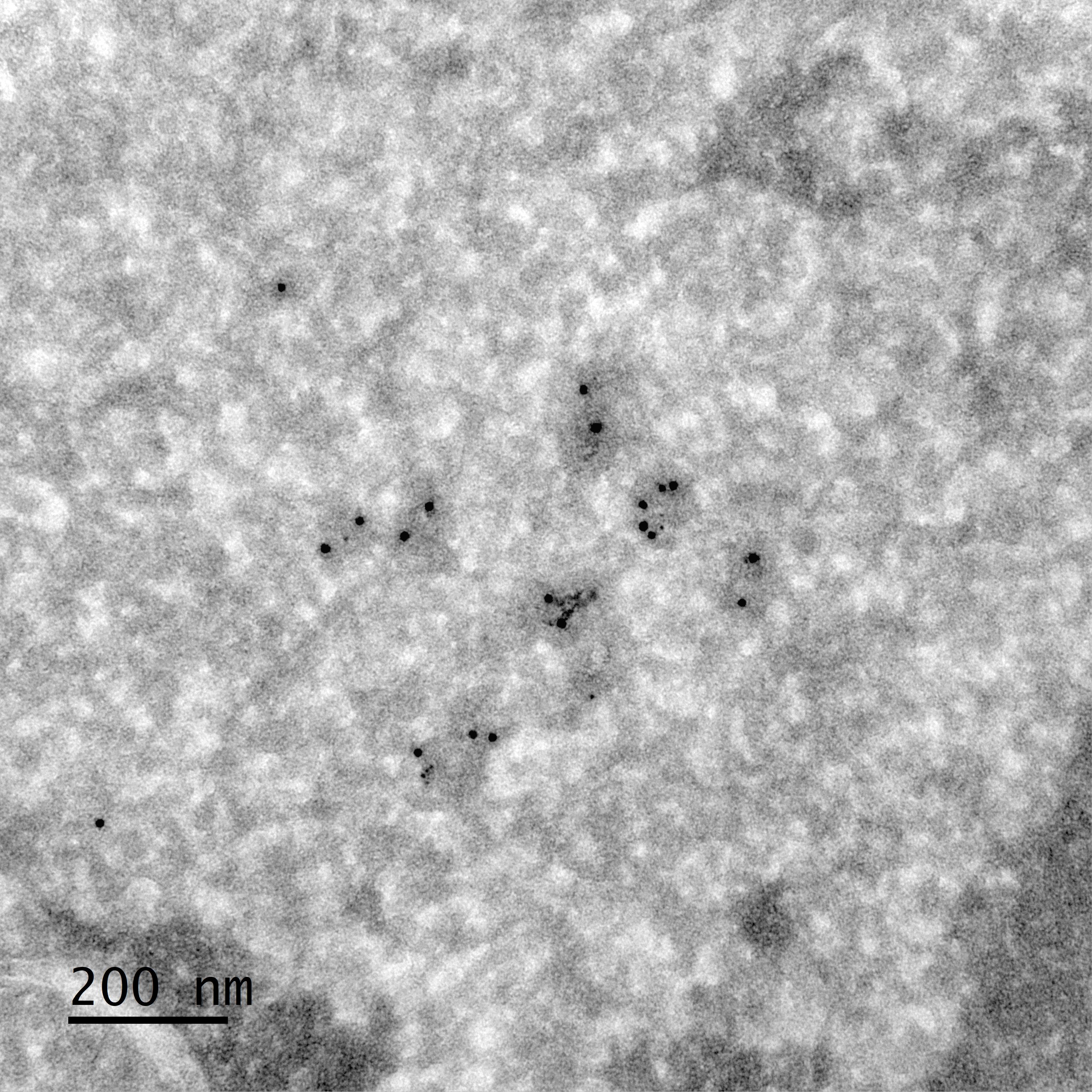

Supplement: Supplementary file 18 — Source data Fig. 4 [file 44318_2024_316_MOESM18_ESM.zip › Figure 4/Fig.4B/1 HIV CA 6nm CPSF6 10nm-0009.jpg]

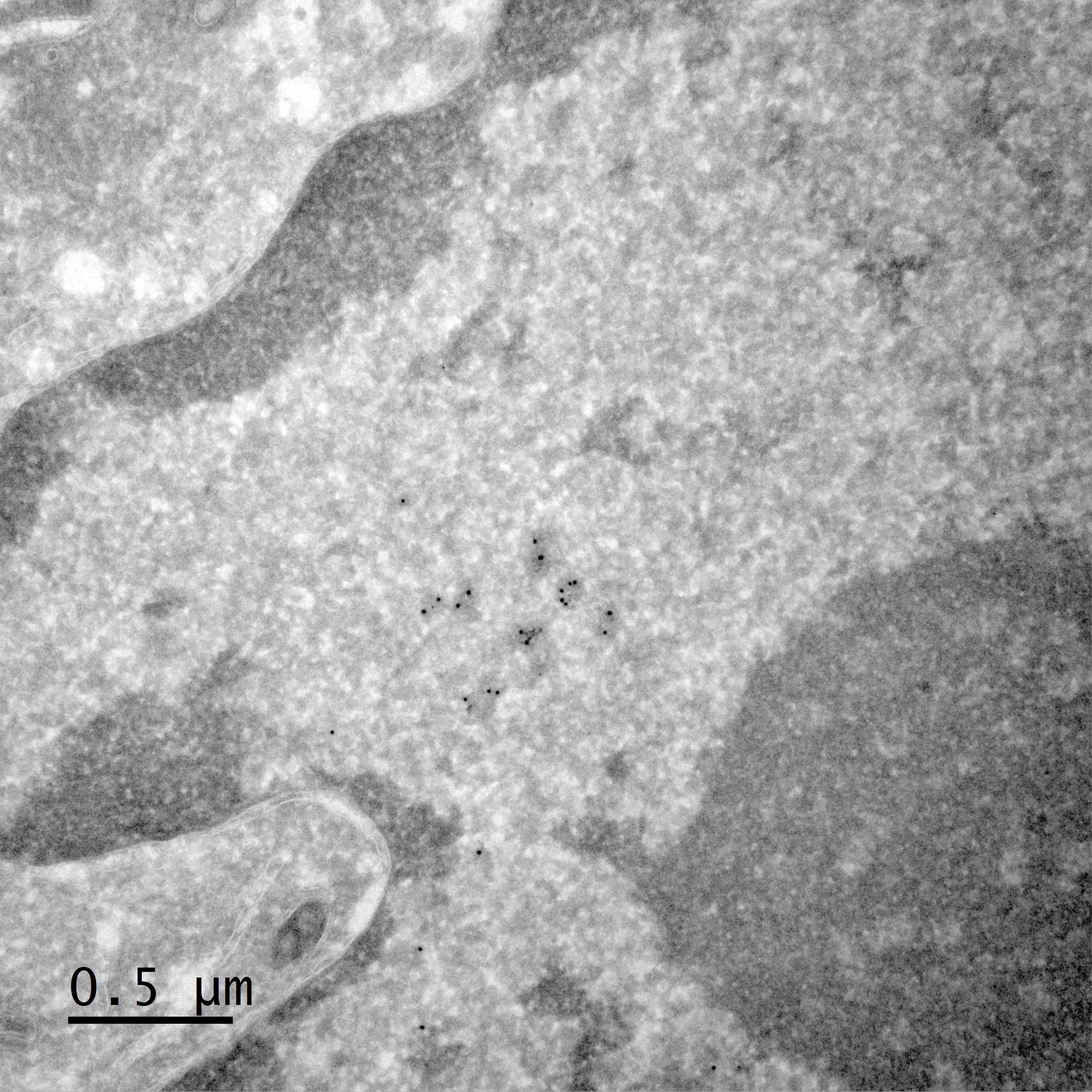

Supplement: Supplementary file 18 — Source data Fig. 4 [file 44318_2024_316_MOESM18_ESM.zip › Figure 4/Fig.4B/1 HIV CA 6nm CPSF6 10nm-0008.jpg]

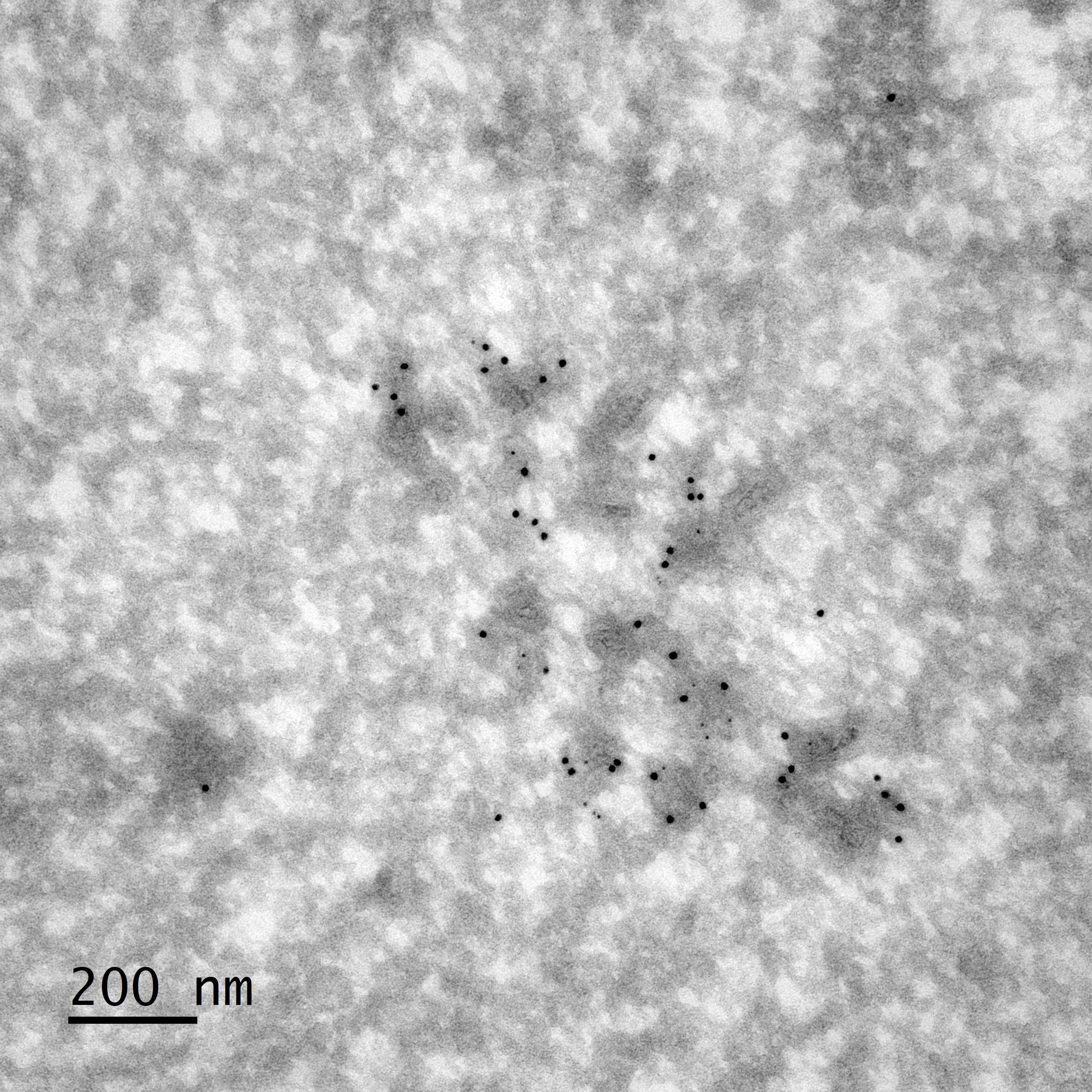

Supplement: Supplementary file 18 — Source data Fig. 4 [file 44318_2024_316_MOESM18_ESM.zip › Figure 4/Fig.4B/1 HIV CA 6nm CPSF6 10nm-0001.jpg]

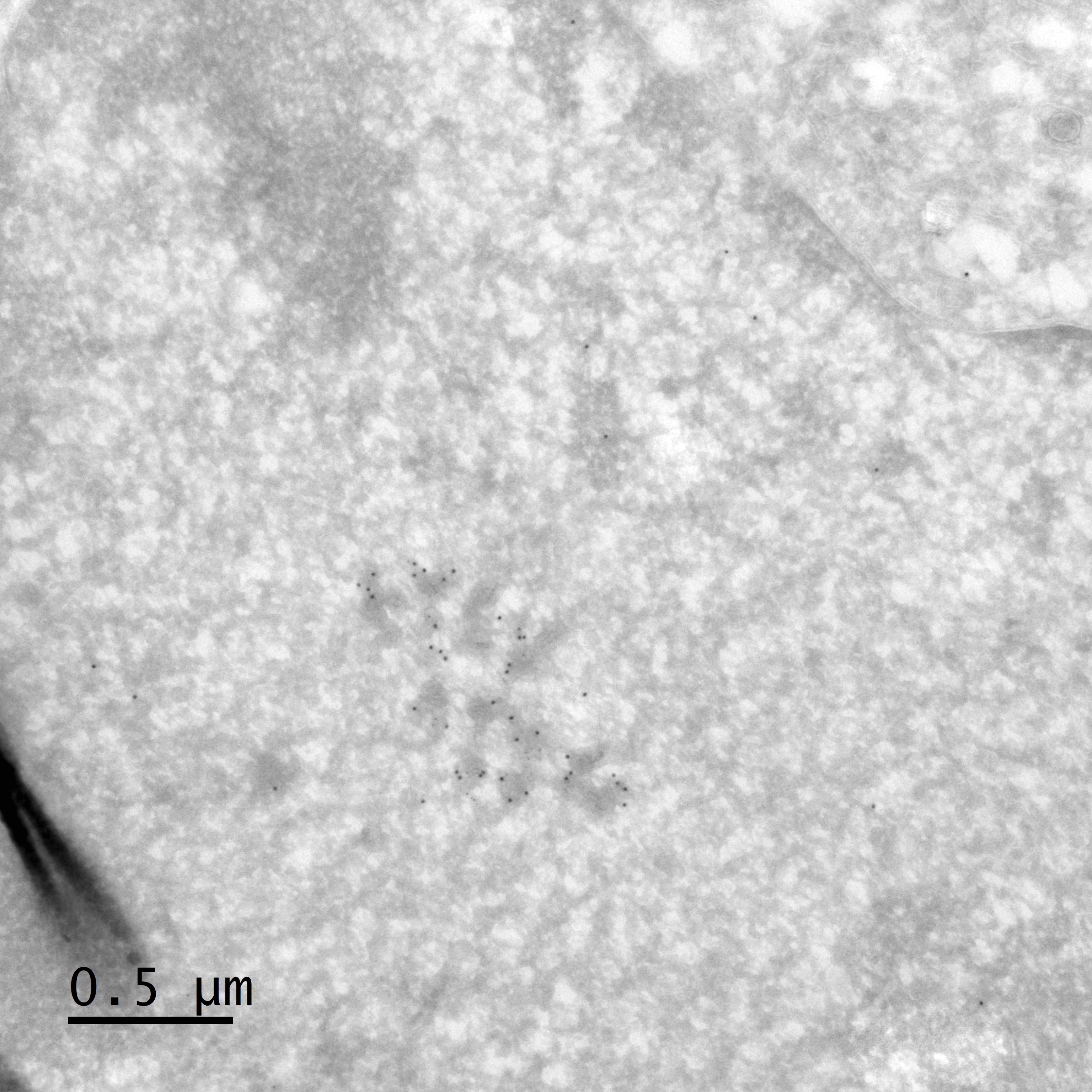

Supplement: Supplementary file 18 — Source data Fig. 4 [file 44318_2024_316_MOESM18_ESM.zip › Figure 4/Fig.4B/1 HIV CA 6nm CPSF6 10nm-0003.jpg]

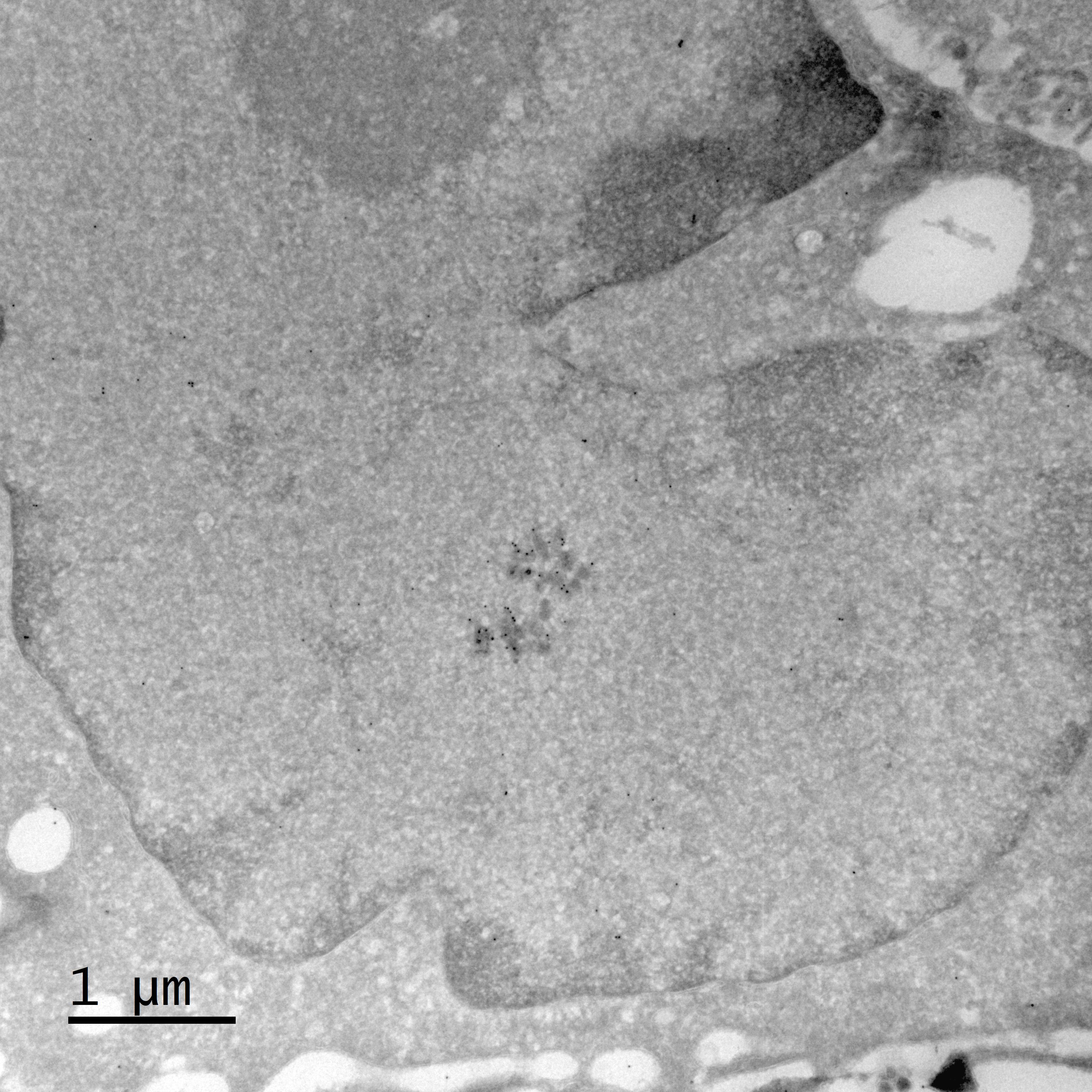

Supplement: Supplementary file 18 — Source data Fig. 4 [file 44318_2024_316_MOESM18_ESM.zip › Figure 4/Fig.4B/1 HIV CA 6nm CPSF6 10nm-0006.jpg]

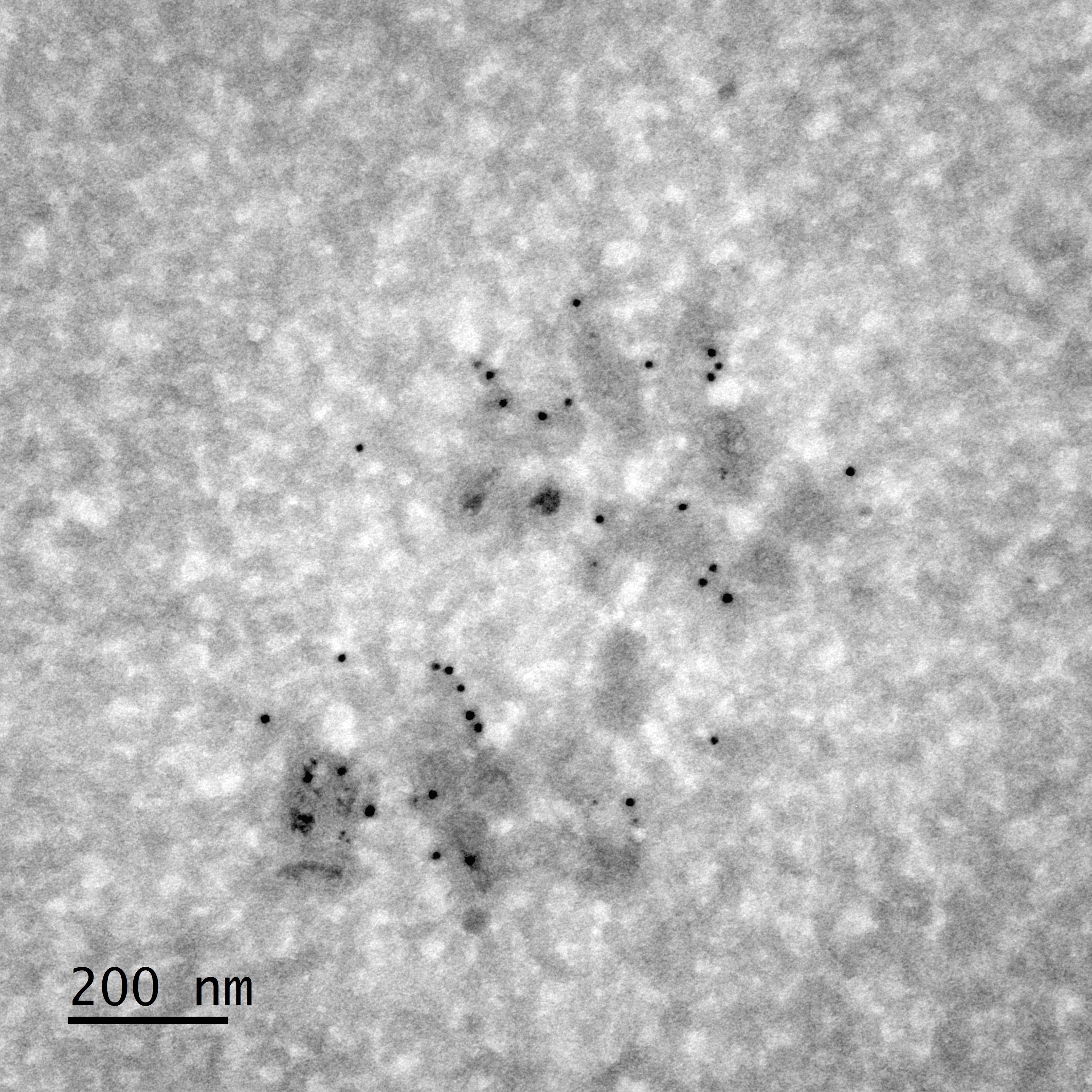

Supplement: Supplementary file 18 — Source data Fig. 4 [file 44318_2024_316_MOESM18_ESM.zip › Figure 4/Fig.4B/1 HIV CA 6nm CPSF6 10nm-0004.jpg]

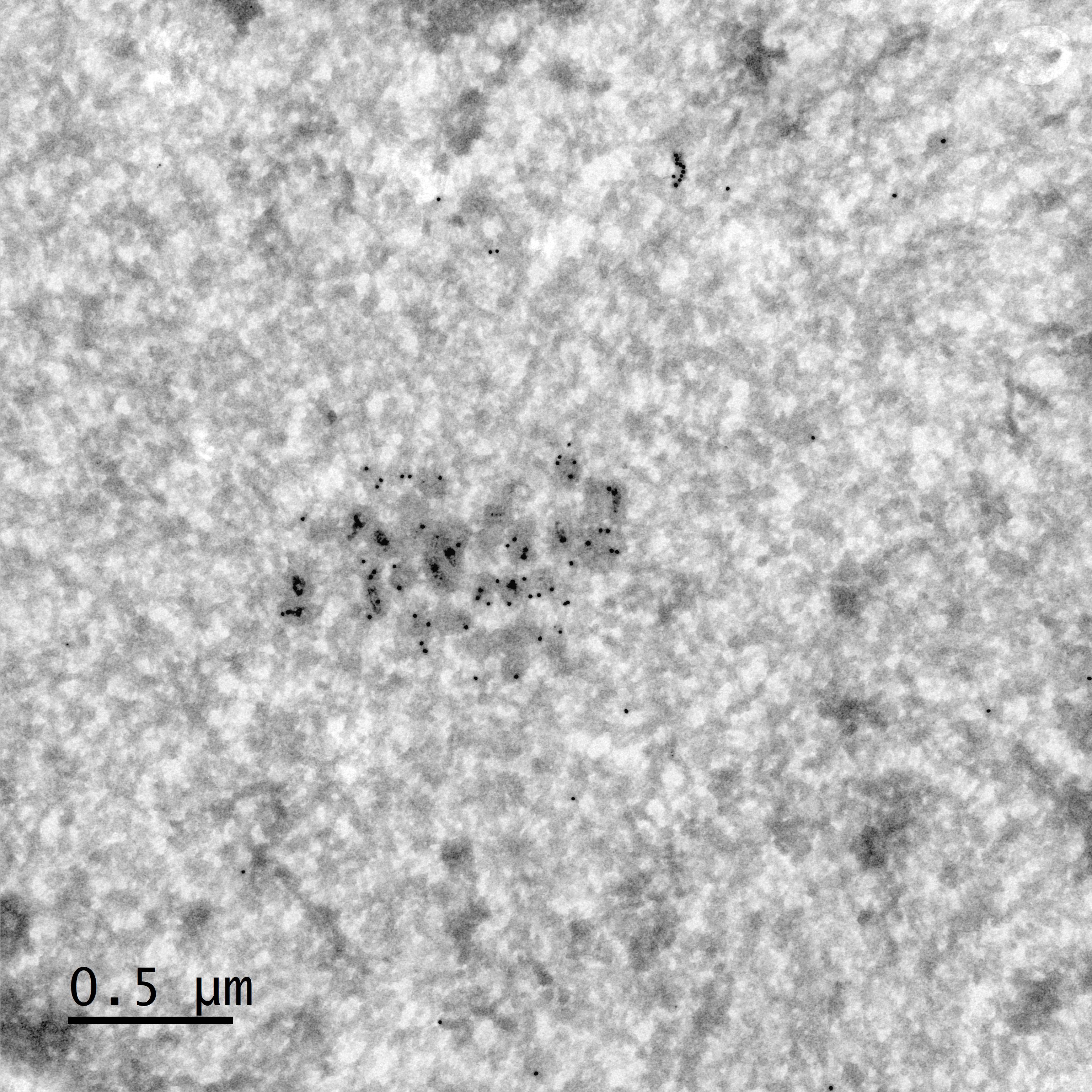

Supplement: Supplementary file 18 — Source data Fig. 4 [file 44318_2024_316_MOESM18_ESM.zip › Figure 4/Fig.4C/2 NEV CA 6nm CPSF6 10nm-0019.jpg]

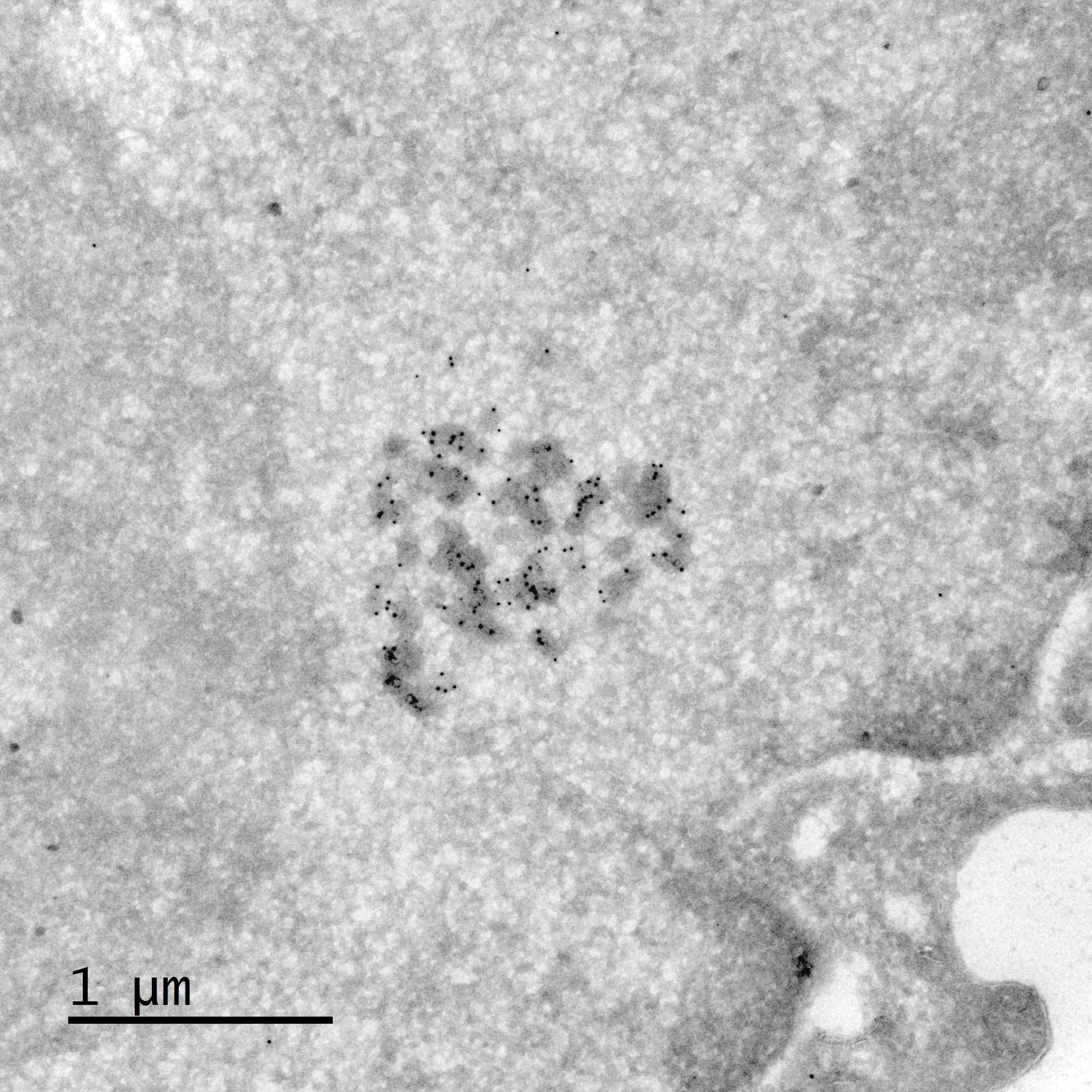

Supplement: Supplementary file 18 — Source data Fig. 4 [file 44318_2024_316_MOESM18_ESM.zip › Figure 4/Fig.4C/2 NEV CA 6nm CPSF6 10nm-0022.jpg]

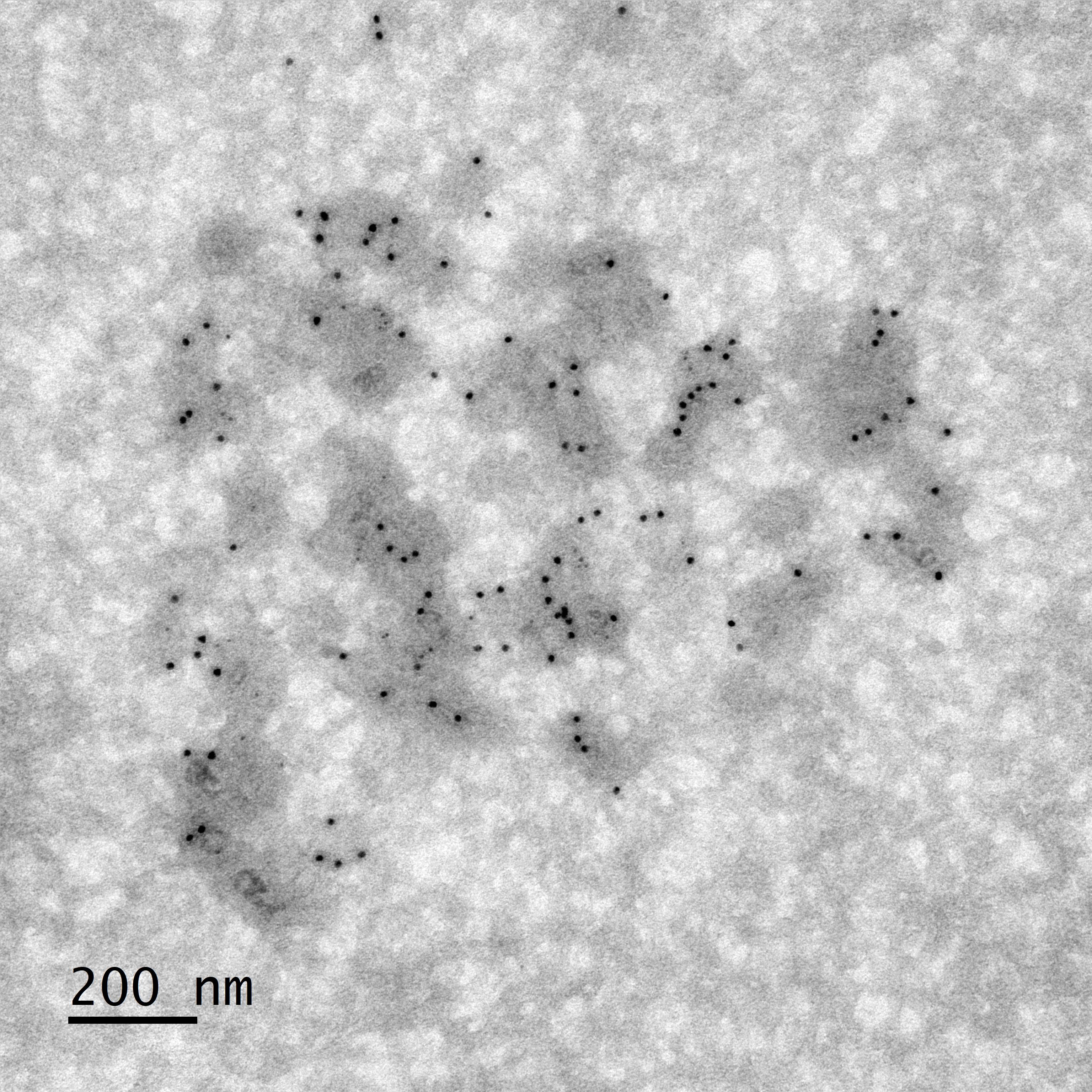

Supplement: Supplementary file 18 — Source data Fig. 4 [file 44318_2024_316_MOESM18_ESM.zip › Figure 4/Fig.4C/2 NEV CA 6nm CPSF6 10nm-0020.jpg]

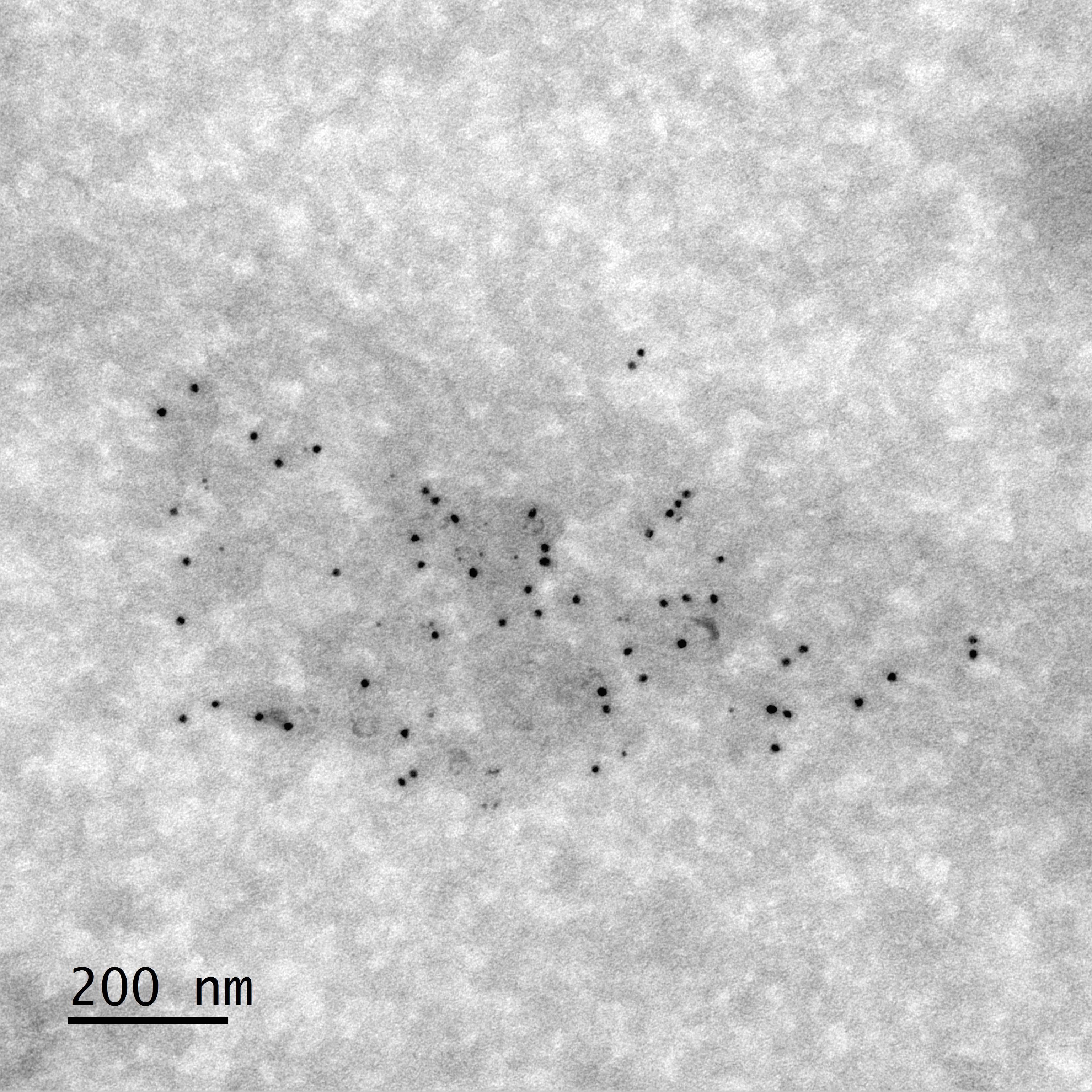

Supplement: Supplementary file 18 — Source data Fig. 4 [file 44318_2024_316_MOESM18_ESM.zip › Figure 4/Fig.4C/2 NEV CA 6nm CPSF6 10nm-0010.jpg]

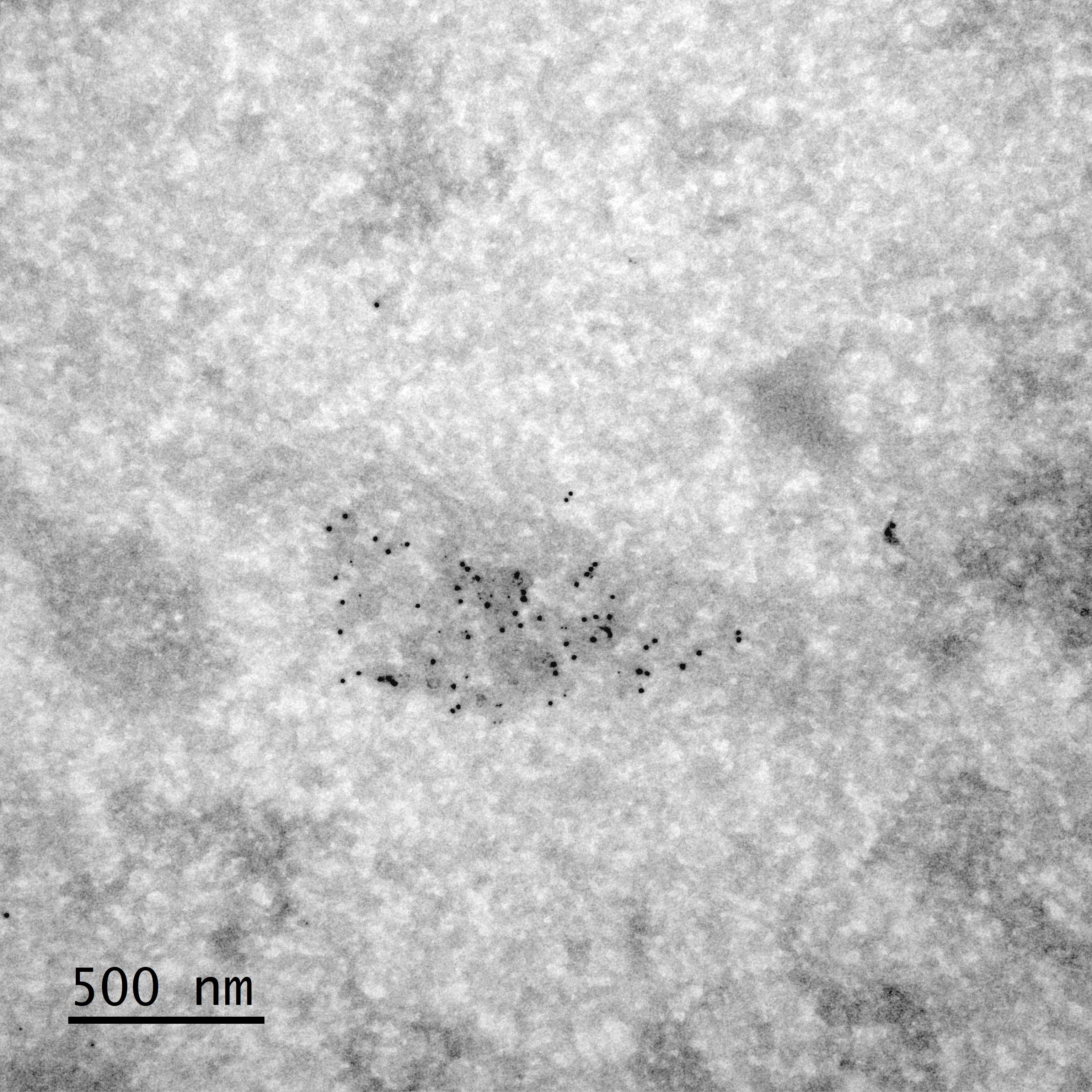

Supplement: Supplementary file 18 — Source data Fig. 4 [file 44318_2024_316_MOESM18_ESM.zip › Figure 4/Fig.4C/2 NEV CA 6nm CPSF6 10nm-0012.jpg]

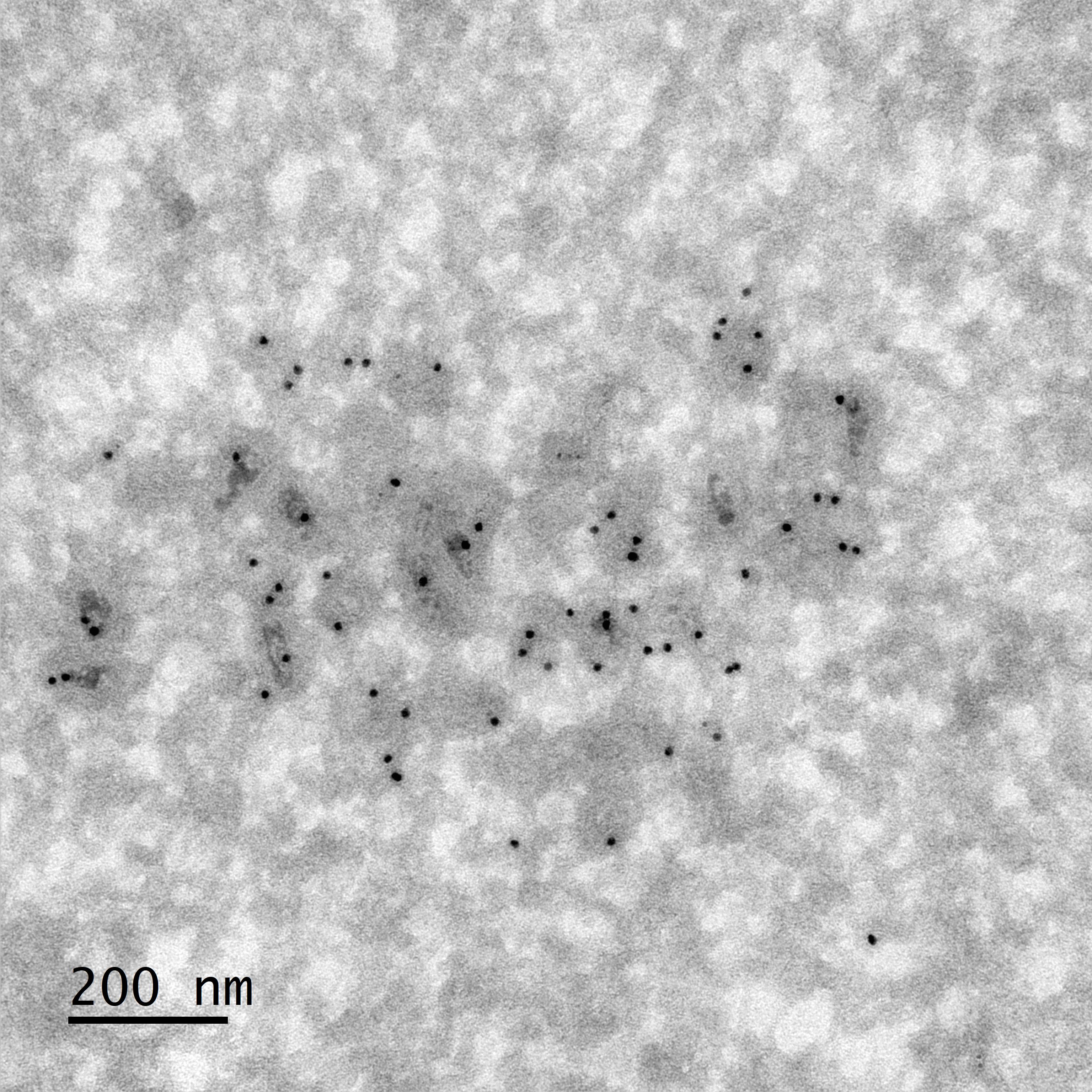

Supplement: Supplementary file 18 — Source data Fig. 4 [file 44318_2024_316_MOESM18_ESM.zip › Figure 4/Fig.4C/2 NEV CA 6nm CPSF6 10nm-0017.jpg]

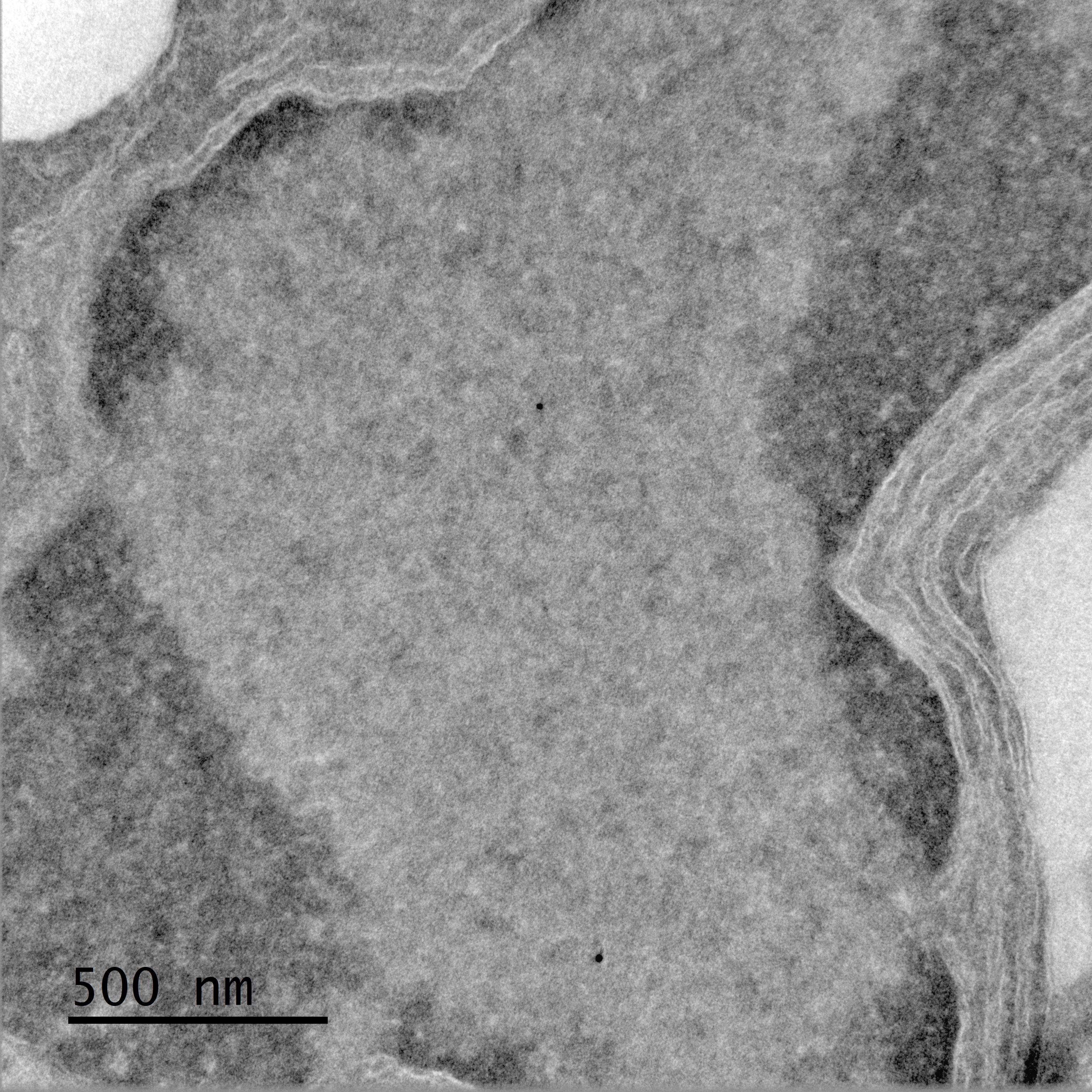

Supplement: Supplementary file 18 — Source data Fig. 4 [file 44318_2024_316_MOESM18_ESM.zip › Figure 4/Fig.4A/Ctrl CA 6nm CPSF6 10nm-0002.jpg]

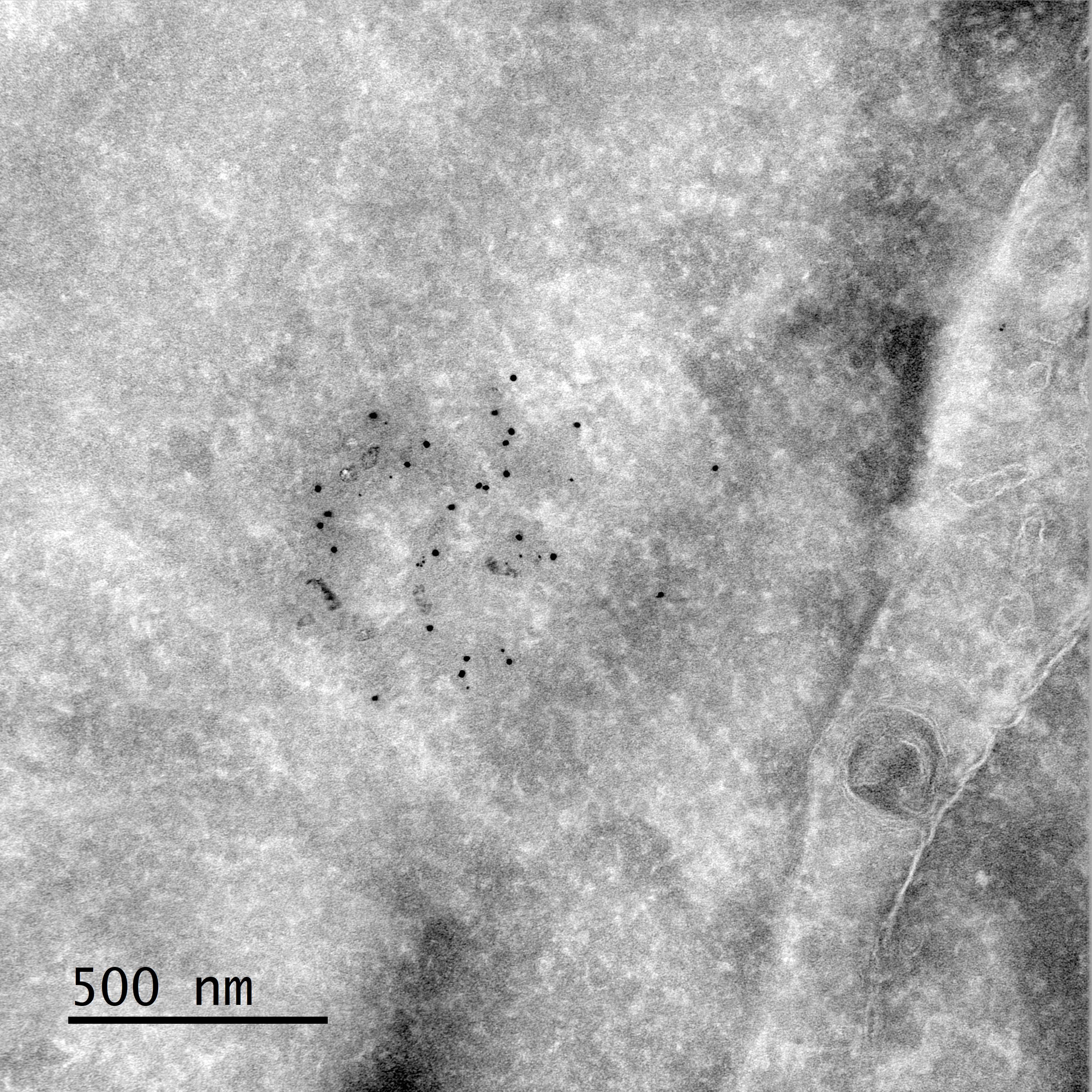

Supplement: Supplementary file 18 — Source data Fig. 4 [file 44318_2024_316_MOESM18_ESM.zip › Figure 4/Fig.4A/HIV CA 6nm CPSF6 10nm-0013.jpg]

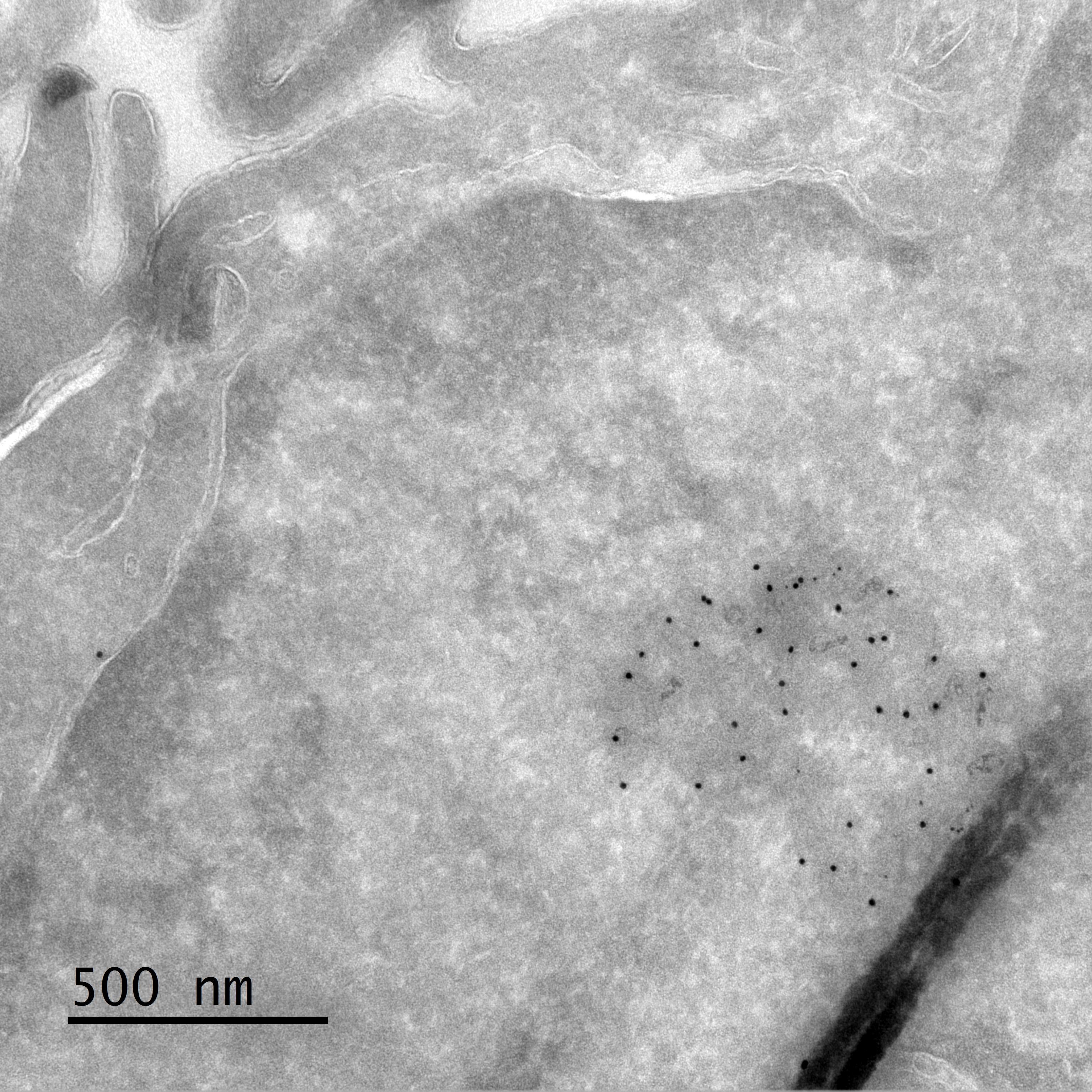

Supplement: Supplementary file 18 — Source data Fig. 4 [file 44318_2024_316_MOESM18_ESM.zip › Figure 4/Fig.4A/HIV CA 6nm CPSF6 10nm-0023.jpg]

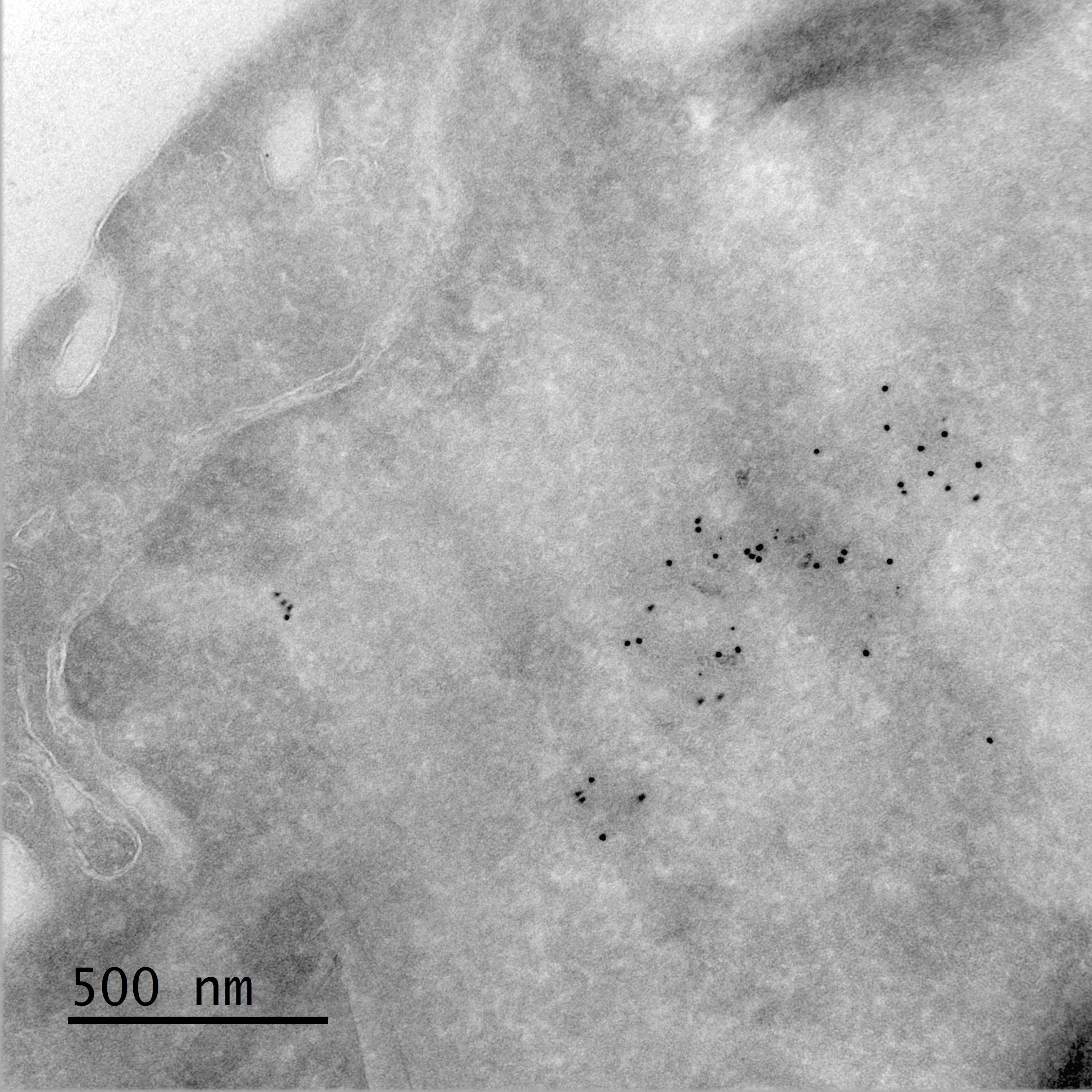

Supplement: Supplementary file 18 — Source data Fig. 4 [file 44318_2024_316_MOESM18_ESM.zip › Figure 4/Fig.4A/HIV CA 6nm CPSF6 10nm-0019.jpg]

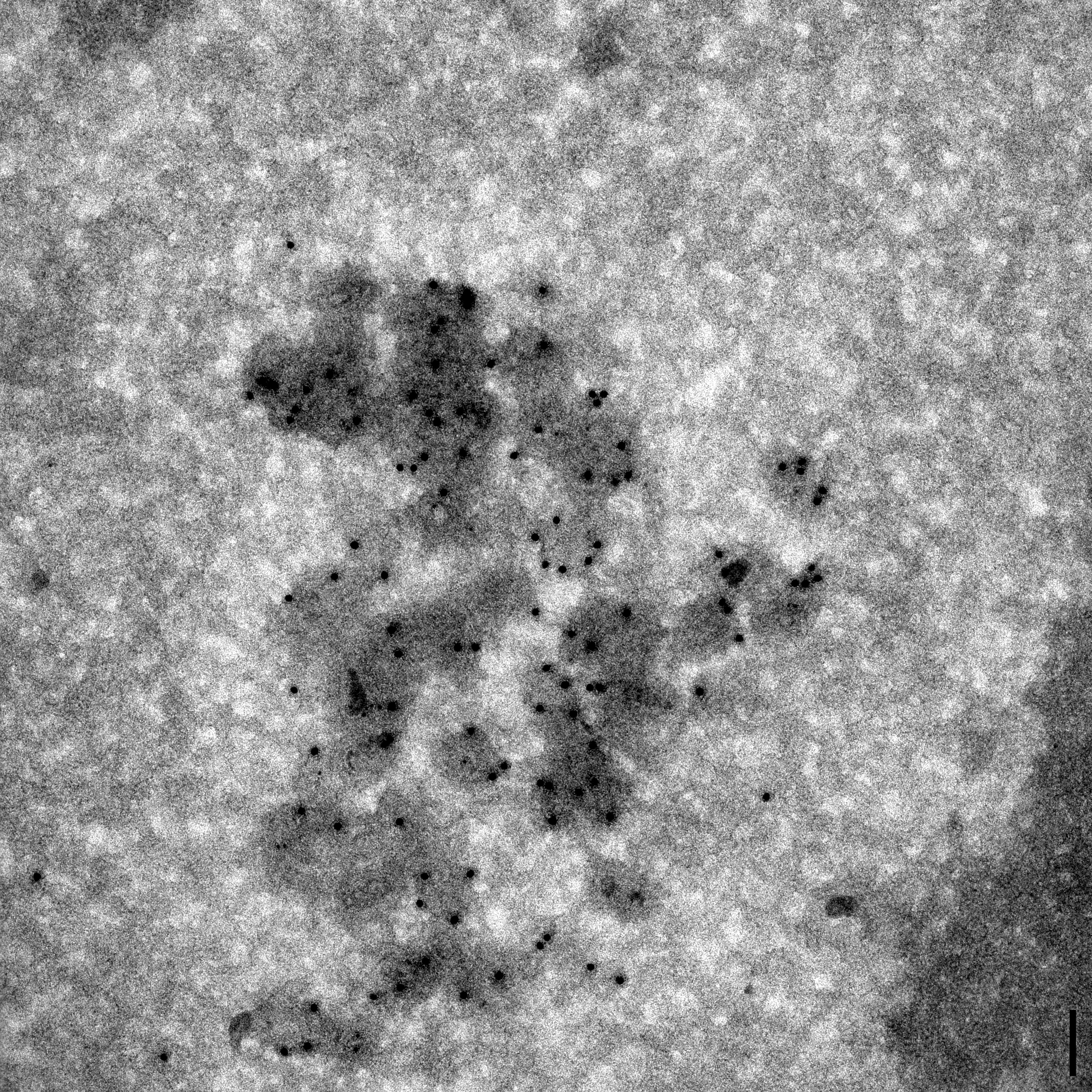

Supplement: Supplementary file 19 — Source data Fig. 5 [file 44318_2024_316_MOESM19_ESM.zip › Figure 5/Figure 5A/5Fa.tif]

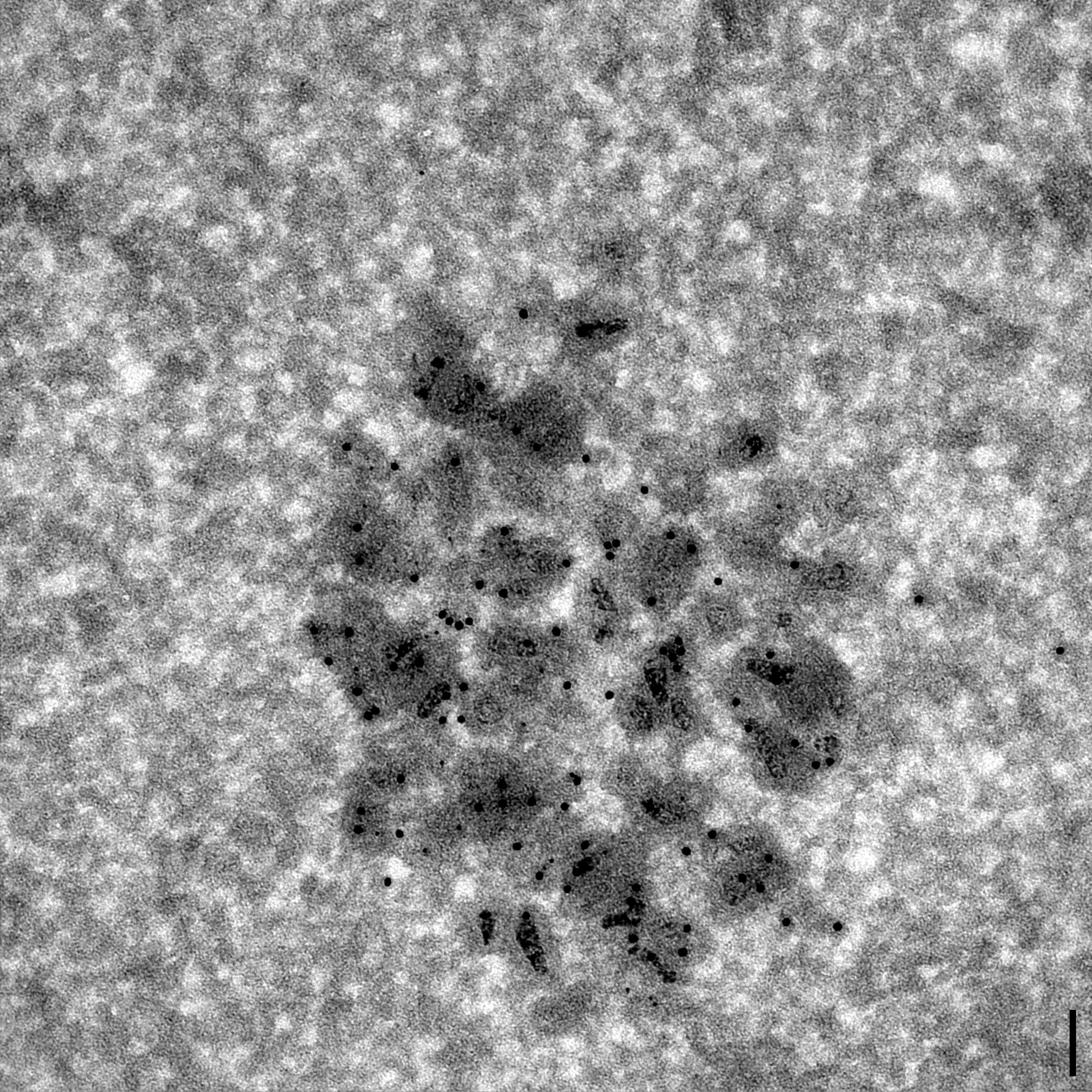

Supplement: Supplementary file 19 — Source data Fig. 5 [file 44318_2024_316_MOESM19_ESM.zip › Figure 5/Figure 5A/5Fg.tif]

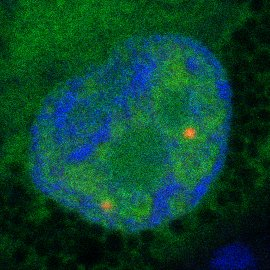

Supplement: Supplementary file 20 — Source data Fig. 6 [file 44318_2024_316_MOESM20_ESM.zip › Figure 6/Figure 6A/HIV1.tiff]

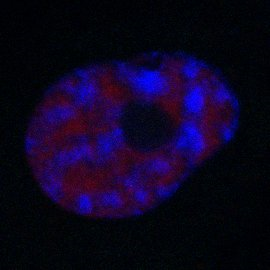

Supplement: Supplementary file 20 — Source data Fig. 6 [file 44318_2024_316_MOESM20_ESM.zip › Figure 6/Figure 6A/HIV1 NEV washout PF74.tiff]

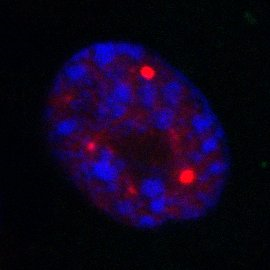

Supplement: Supplementary file 20 — Source data Fig. 6 [file 44318_2024_316_MOESM20_ESM.zip › Figure 6/Figure 6A/HIV1 NEV.tiff]

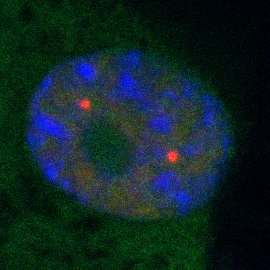

Supplement: Supplementary file 20 — Source data Fig. 6 [file 44318_2024_316_MOESM20_ESM.zip › Figure 6/Figure 6A/HIV1 NEV washout.tiff]

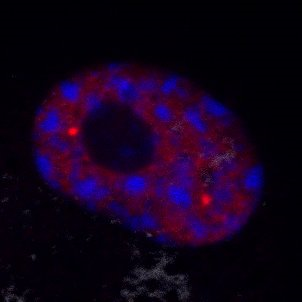

Supplement: Supplementary file 20 — Source data Fig. 6 [file 44318_2024_316_MOESM20_ESM.zip › Figure 6/Figue 6 C/NO PF74.tiff]

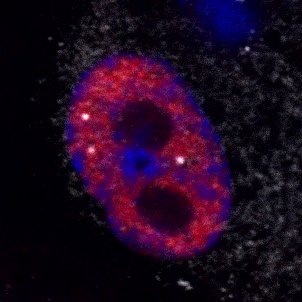

Supplement: Supplementary file 20 — Source data Fig. 6 [file 44318_2024_316_MOESM20_ESM.zip › Figure 6/Figue 6 C/PF74.tiff]

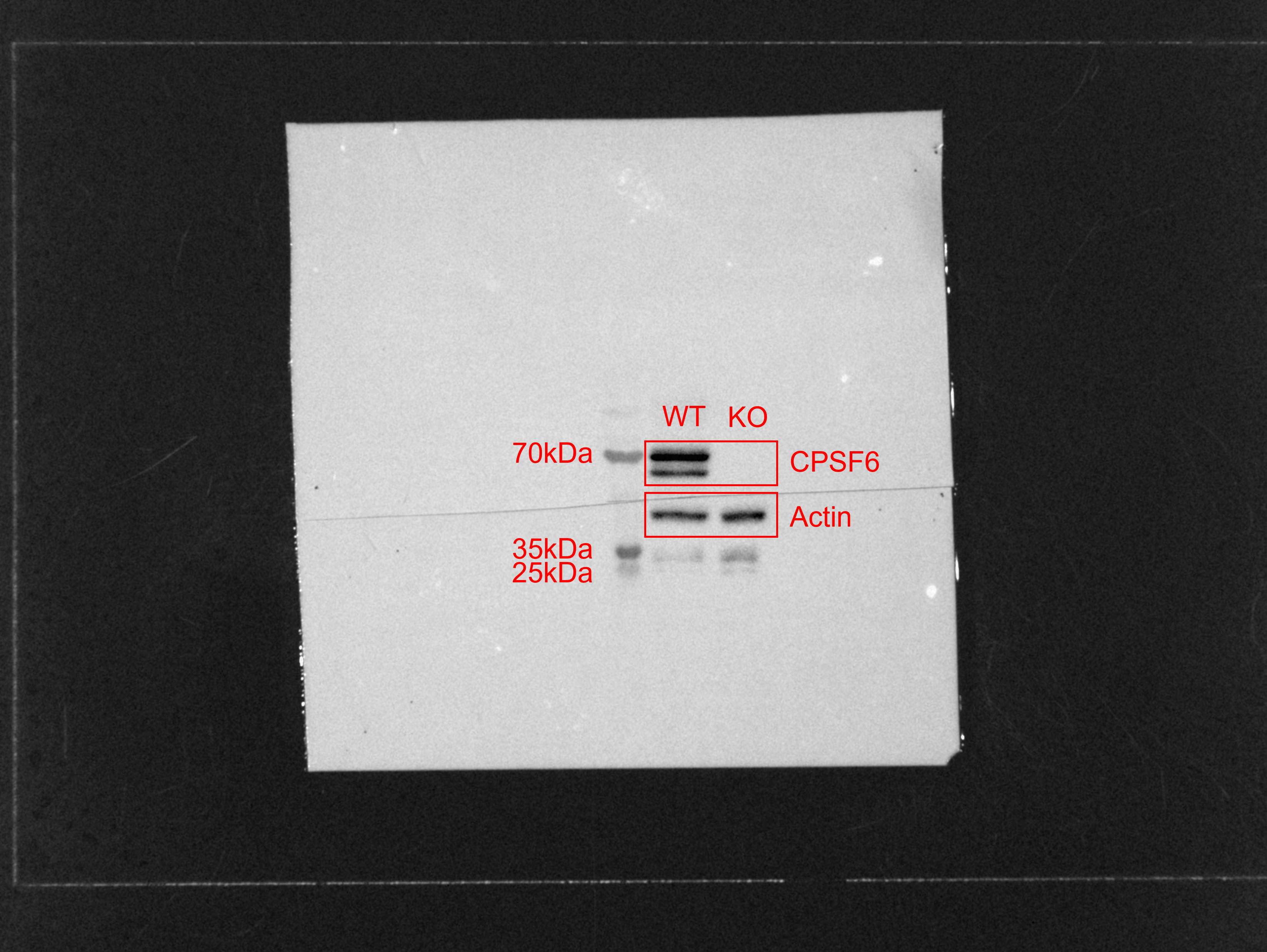

Supplement: Supplementary file 21 — Source data Fig. 7 [file 44318_2024_316_MOESM21_ESM.zip › Figure 7/Figure 7D/CPSF6 western blot.tif]

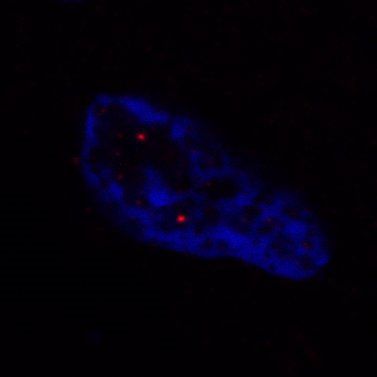

Supplement: Supplementary file 21 — Source data Fig. 7 [file 44318_2024_316_MOESM21_ESM.zip › Figure 7/Figure 7A/Infected Nevirapine.tiff]

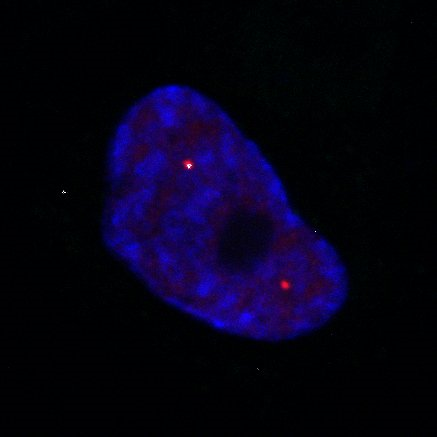

Supplement: Supplementary file 21 — Source data Fig. 7 [file 44318_2024_316_MOESM21_ESM.zip › Figure 7/Figure 7A/Infected No drug.tiff]
