# Supplementary material for: In vivo HIV-1 nuclear condensates safeguard against cGAS and license reverse transcription
Source: EMBO J. 2024 Dec 2;44(1):166–99. doi: 10.1038/s44318-024-00316-w (PMC11697293; doi:10.1038/s44318-024-00316-w)
Supplement: Supplementary file 21 — Source data Fig. 7 [file 44318_2024_316_MOESM21_ESM.zip › Figure 7/Figure 7B/Table Figure 7B.docx]

| **Exp. 1** | Sample name | Replicate 1(Mean O/D) | Replicate 2 (Mean O/D) | Fold induction  repl1/NI (Concentration (pmol/ng)/normalized) | Fold induction repl2/NI  (Concentration (pmol/ng)/normalized) |
| --- | --- | --- | --- | --- | --- |
|  | NI | 1.055 | 1.125 | 1 | 1 |
|  | Infected | 1.073 | 1.098 | 1.017061611 | 0.976 |
|  | Infected+NEV | 1.167 | 1.126 | 1.106161137 | 1.000888889 |
|  | Infected+PF74 | 0.846 | 0.812 | 2.74479341 | 4.53634341 |
|  |  |  |  |  |  |
| **Exp.2** | Sample name | Replicate 1(Mean O/D) | Replicate 2 (Mean O/D) | Fold induction  repl1/NI (Concentration (pmol/ng)/normalized) | Fold induction repl2/NI  (Concentration (pmol/ng)/normalized) |
|  | NI | 1.226 | 1.141 | 1 | 1 |
|  | Infected | 0.938 | 1.004 | 2.617162396 | 1.914189829 |
|  | Infected+NEV | 1.148 | 1.184 | 0.967358832 | 0.815623939 |
|  | Infected+PF74 | 0.8 | 0.883 | 5.033557577 | 3.396556339 |
|  |  |  |  |  |  |
| **Exp.3** | Sample name | Replicate 1(Mean O/D) | Replicate 2 (Mean O/D) | Fold induction  repl1/NI (Concentration (pmol/ng)/normalized) | Fold induction repl2/NI  (Concentration (pmol/ng)/normalized) |
|  | NI | 1.171 | 1.213 | 1 | 1 |
|  | Infected | 1.203 |  | 0.946340314 |  |
|  | Infected | 1.166 | 1.188 | 1.122241428 | 1.014043163 |
|  | Infected+PF74 | 0.92464905 | 0.69165805 | 5.814781493 | 4.349585826 |
