# Supplementary material for: In vivo HIV-1 nuclear condensates safeguard against cGAS and license reverse transcription
Source: EMBO J. 2024 Dec 2;44(1):166–99. doi: 10.1038/s44318-024-00316-w (PMC11697293; doi:10.1038/s44318-024-00316-w)
Supplement: Supplementary file 23 — Expanded View Figures [file 44318_2024_316_MOESM23_ESM.pdf]

Expanded View Figures

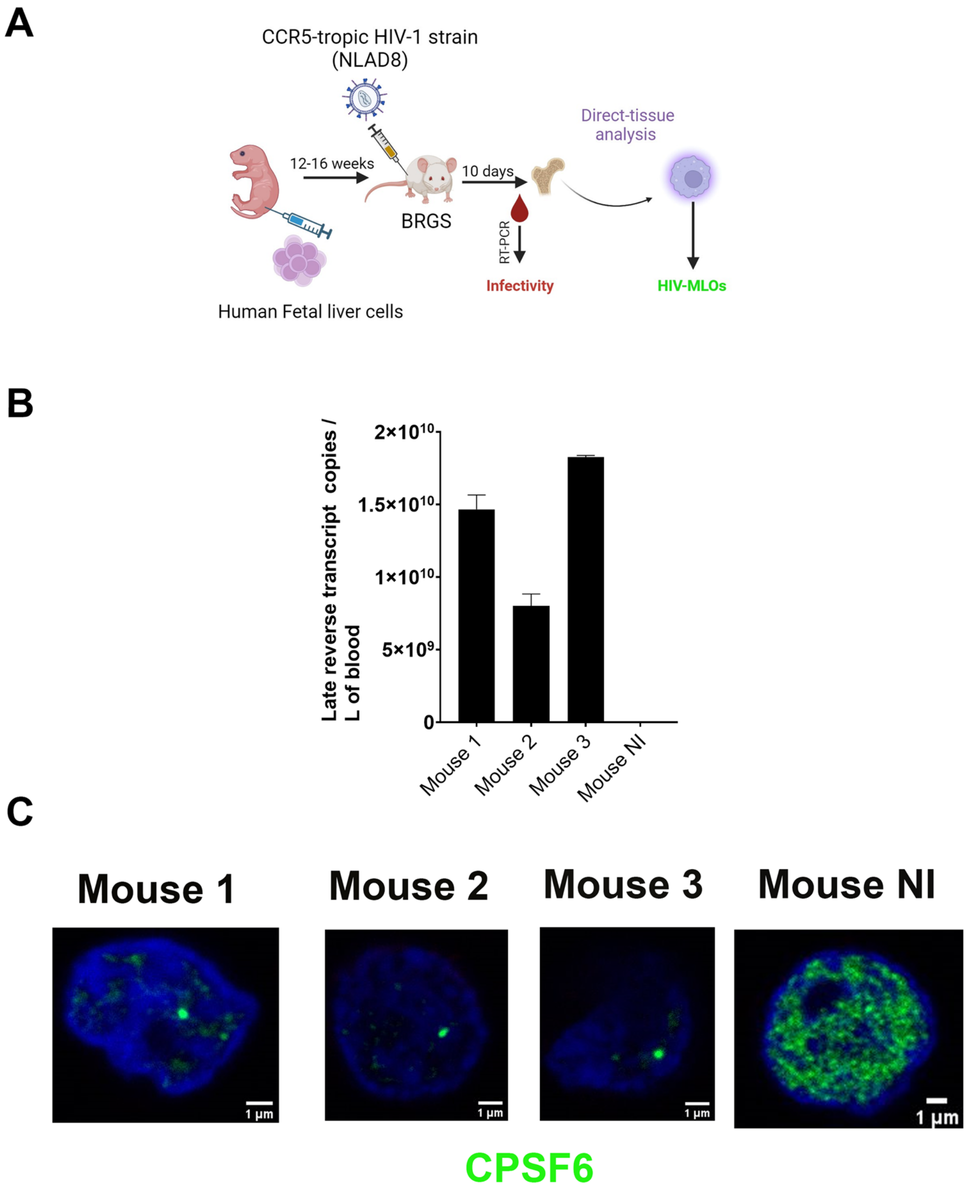

Figure EV1. HIV-1-MLOs build during in vivo infection, related to Fig. 3.

(**A**) Schema of CCR5-tropic HIV-1 strain (NLAD8) infection in BRGS mice for 10 days. Bone marrow human CD4+ cells were immediately labeled and imaged without the need of cell culture passage. (**B**) Graph showing viral infectivity in BRGS mice by quantitative RT PCR (Data are shown as the mean ± SD of two replicates). (**C**) CD4+ cells derived from BM of infected BRGS mice were directly fixed and labeled for the detection of CPSF6 (in green). Nuclei were stained with Hoechst (in blue).

© The Author(s)

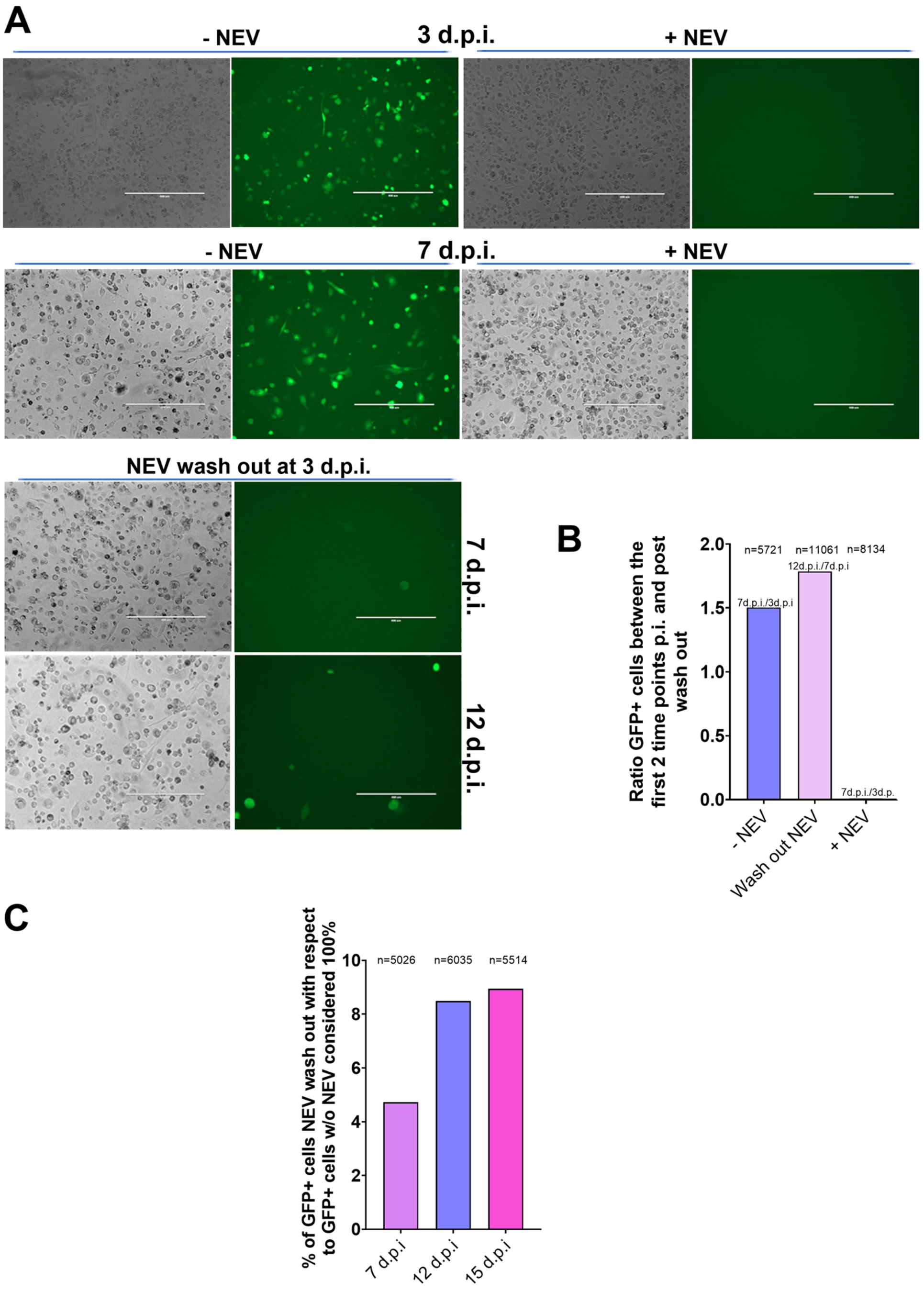

Figure EV2. Asynchronous viral reactivation in nuclear HIV-1-MLOs, related to Fig. 2 and 4.

(**A**) THP-1 cells infected with HIV-1 ΔEnv pseudotyped with VSV-G and carrying the GFP as reporter gene at 3 and 7 d.p.i. +/− NEV or after washout of NEV at 3 d.p.i. and live imaging (EVOS microscope) were done at 7 and 12 d.p.i., respectively. (**B**) The increased ratio of GFP+ cells between the first 2 time points p.i. and post wash out. (**C**) Percentage of GFP after recovery of RT post wash out at different time p.i. Cells infected without NEV were considered as 100%.

© The Author(s)

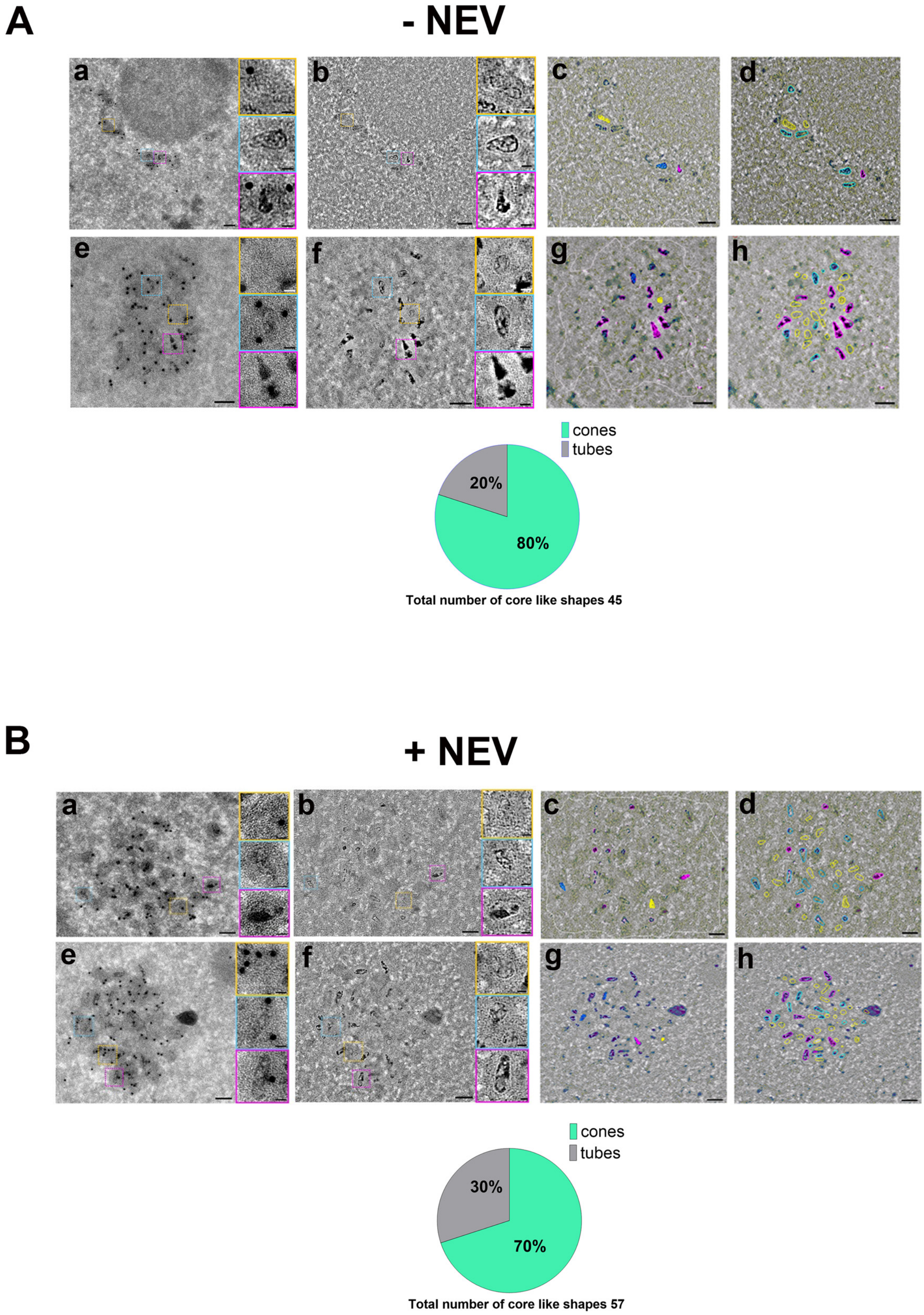
A
- NEV
a
b
c
d
e
f
g
h
cones
tubes
20%
80%
Total number of core like shapes 45
B
+ NEV
a
b
c
d
e
f
g
h
cones
tubes
30%
70%
Total number of core like shapes 57

Figure EV3. Viral core classification in HIV-1-MLOs, related to Fig. 5.

(**A**) Nuclear HIV-1-MLOs composition classes in absence of NEV: in the panels (a, e) projection images at sites of interest as recorded by the detector. Immunogold labeling (10 nm beads) are labeling the CPSF6 at HIV-1-MLOs. In the panels (b, f) a slice from the middle of the dual-axis tomographic volume where HIV cores are more easily observed. Examples of the three different classes as they identified according to the criteria below in dense cores (in magenta boxes), lighter cores (in blue boxes) and ghosts (in yellow boxes). Assigning core classes in absence of NEV: Pixel classification predictions for two different sites shown here (c, d, g, h). In the left column, initial user input (labels) are shown in the images as thin lines together with the predictions for the rest of the image (c, g). The prediction of ghosts was not possible using all features selected in Ilastik, and the pixels were most commonly classified as background (background was seeded as a separate longer label, here in white). In the right column (d, h), pixel classification predictions are overlaid with the manual traced contours of cores, colored according to the underline predictions. Magenta and blue predictions outside the cores are either due to immunogold beads that are still contributing to the slice under evaluation, or sometimes due to Tokuyasu artifacts (darker/thicker area in Bg). Scale bars = 100 nm. Percentage of HIV core shapes (pie chart): HIV cores were included in this analysis only when their longer axis was parallel or near parallel to the tomographic XY plane. The cores were classified as cones in the case an orientation (head to tip) could be assigned easily to them. The characteristic conical shape of the HIV was easily identifiable in most of the cases. In the rest of the cases, where the head could not be identified, HIV cores were assigned as of tubular shape. (**B**) Nuclear HIV-1-MLOs composition in presence of NEV (panels a, b, e, f), assigning core classes (panels c, d, g, h) and percentage of HIV core shapes (pie chart) have been processed as in (**A**).

 © The Author(s)

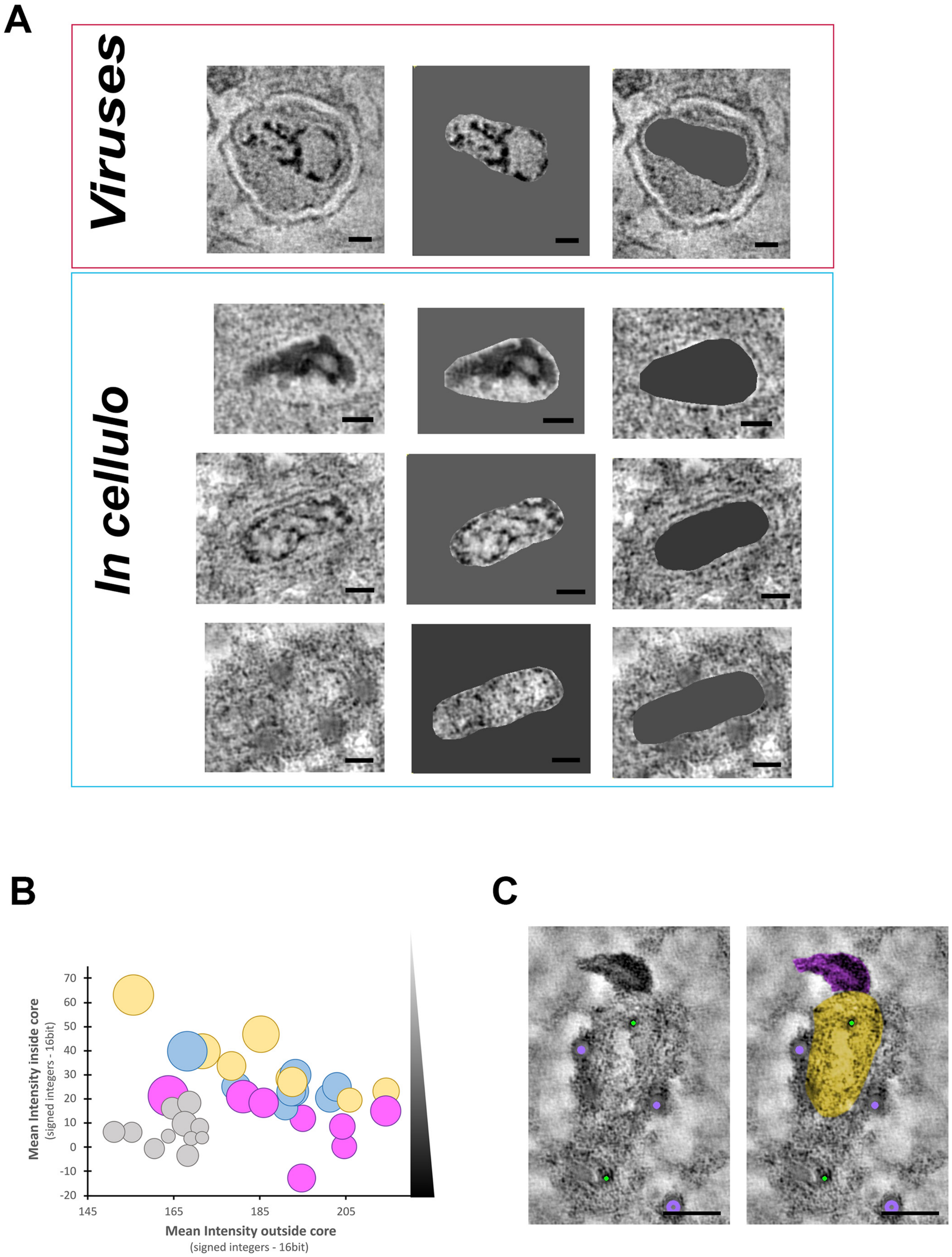
A
Viruses
In cellulo
B
70
60
50
40
30
20
10
0
-10
-20
145
165
185
205
Mean Intensity inside core
(signed integers - 16bit)
Mean Intensity outside core
(signed integers - 16bit)
C

Figure EV4. Viral core intensity analysis and comparison to free viruses, related to Fig. 5.

(**A**) Intensity analysis of HIV cores. A representative core from mature virions before infection (first row), a representative of a dense core inside a nuclear MLO (second row), a representative core with lighter dark areas inside a nuclear MLO (third row) and a representative of a "ghost" or empty core in HIV-1-MLOs (forth row). In middle column only the inside of the core intensities are maintained, while in the third column only the exterior intensities are visible. (**B**) A scatter plot from the internal mean intensity (y axes) (negative values represent denser signal) vs external mean intensity (x axes) of individual cores. The diameter of the dots represents the standard deviation of the mean interior intensity for each individual core. In gray, the corresponding values from mature virions before the infection, in magenta the values from full cores inside the MLOs, cores in MLOs with intermediate intensities are in blue and the values from ghosts in MLOs are in yellow. (**C**) A ghost core detected by anti-CA antibody (green bead) is decorated with anti-CPSF6 antibody (purple beads). The left panel shows the original volume section, while the right panel is pseudocolored: yellow indicates the ghost core, and purple highlights the dark intensity near the core's head. Scale bar 50 nm.

© The Author(s)

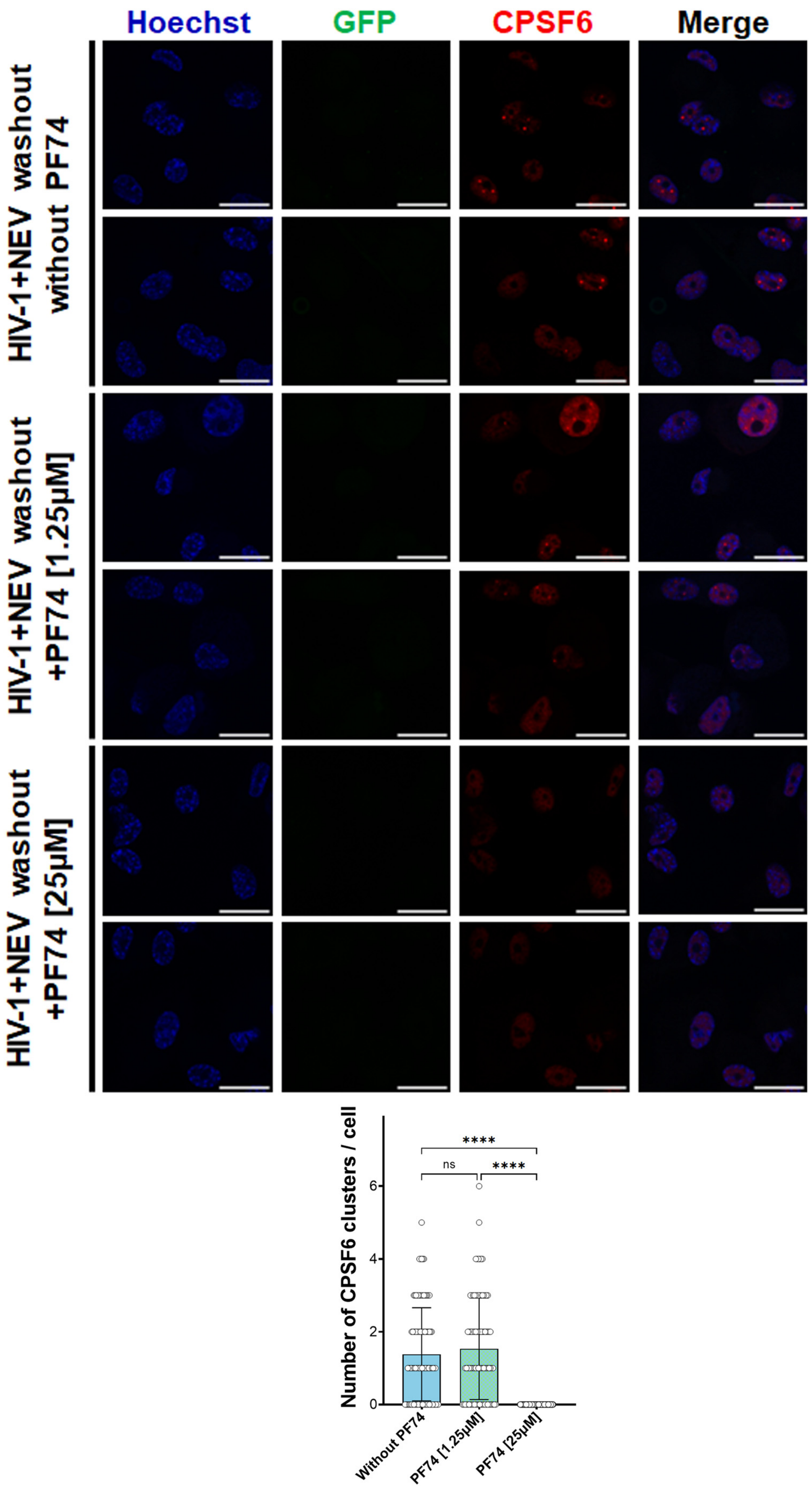
Hoechst
GFP
CPSF6
Merge
HIV-1+NEV washout without PF74
HIV-1+NEV washout +PF74 [1.25µM]
HIV-1+NEV washout +PF74 [25µM]
Number of CPSF6 clusters / cell
0
2
4
6
ns
****
****
Without PF74
PF74 [1.25µM]
PF74 [25µM]

Figure EV5. High PF74 dosage leads to the disassembly of CPSF6 condensates, related to Figs. 6, 7 and 8.

Immunostaining of cells kept in the same culture conditions as in Fig. 6A and an additional condition with lower PF74 dosage (1.25 µM) (blue = Hoechst; green = GFP; red = CPSF6; scale bar = 20 µm) (top panel). Graph showing the quantification of CPSF6 clusters per cell in cells treated with PF74 (1.25 and 25 µM) or not (Data are shown as the mean ± SD of two datasets of biological replicates, statistical test: ordinary one-way ANOVA; ****: *p*-value= 6×10^{-14}; ns: *p*-value = 0.6040) (bottom panel).

 © The Author(s)
